# Long-lived Paleoproterozoic eclogitic lower crust

**DOI:** 10.1038/s41467-021-26878-5

**Published:** 2021-11-12

**Authors:** Sebastian Buntin, Irina M. Artemieva, Alireza Malehmir, Hans Thybo, Michal Malinowski, Karin Högdahl, Tomasz Janik, Stefan Buske

**Affiliations:** 1 Department of Earth Sciences, Uppsala University, Uppsala, Sweden; 2 Department of Geophysics, Stanford University, Stanford, CA USA; 3 Marine Geodynamics Section, GEOMAR Helmholtz Center for Ocean Research, Kiel, Germany; 4 School of Earth Sciences, China University of Geosciences, Wuhan, China; 5 Eurasia Institute of Earth Sciences, Istanbul Technical University, Istanbul, Turkey; 6 Institute of Geophysics, Polish Academy of Sciences, Warsaw, Poland; 7 Technical University Bergakademie Freiberg, Freiberg, Germany

**Keywords:** Geodynamics, Geophysics, Seismology

## Abstract

The nature of the lower crust and the crust-mantle transition is fundamental to Earth sciences. Transformation of lower crustal rocks into eclogite facies is usually expected to result in lower crustal delamination. Here we provide compelling evidence for long-lasting presence of lower crustal eclogite below the seismic Moho. Our new wide-angle seismic data from the Paleoproterozoic Fennoscandian Shield identify a 6–8 km thick body with extremely high velocity (Vp ~ 8.5–8.6 km/s) and high density (>3.4 g/cm^3^) immediately beneath equally thinned high-velocity (Vp ~ 7.3–7.4 km/s) lowermost crust, which extends over >350 km distance. We relate this observed structure to partial (50–70%) transformation of part of the mafic lowermost crustal layer into eclogite facies during Paleoproterozoic orogeny without later delamination. Our findings challenge conventional models for the role of lower crustal eclogitization and delamination in lithosphere evolution and for the long-term stability of cratonic crust.

## Abstract (web summary)

The nature of the lower crust and the crust-mantle transition is fundamental to Earth Sciences. Here, the authors provide evidence for long-lasting presence of lower crustal eclogite below the seismic Moho, challenging conventional models.

## Introduction

The fundamental questions of secular change in crustal growth rate^1,2^ and crustal recycling^3,4^ as well as a long-term stability of cratonic lithosphere^5^ are closely linked to crust–mantle interaction. The continental crust varies in composition from felsic to mafic rocks, and the amount of mafic materials in the lower crust and its composition are for long debated^6–9^. Minimum estimates require the lower crust to include 10–20% of mafic materials^8^ and, typically, it is expected to be 80% mafic with dominating granulite-facies rocks^6^. The immediately underlying upper (lithospheric) mantle has at least 10–20% of ultramafic peridotitic rocks, while mafic rocks may still be present^10^.

Mafic rocks have lower seismic velocities and are less dense than ultramafic rocks^11,12^, which commonly results in significant differences in physical properties of the lower crust and the upper mantle, and results in a seismically detectable crust–mantle transition named the Moho discontinuity. However, metamorphic reactions and compositional heterogeneity in the lower crust and upper mantle may regionally reduce the velocity and density contrast across the Moho boundary. In nearly all cases, the continental Moho is inaccessible for direct observations and the composition of the lower crust and the nature of the continental crust–mantle transition are largely known only from spatially restricted xenolith sampling^6,10^.

Granulite-facies rocks are prone to transformation to eclogite, although the reaction rate cannot be constrained in laboratory experiments and the conditions required for formation of abundant eclogite-facies rocks are debated. Conventional models for the gabbro to eclogite transition to occur within geological time in cool cratonic lithosphere require *T* > 600–800 °C and the presence of a fluid phase to promote element mobility^13–15^; the requirement for fluid infiltration has been documented in rock outcrops and xenolith studies^16–18^. Physical properties of eclogites depend on the composition of the mother rock that has undergone phase transformation and on transformation degree^19^, and they are distinctly different from the lower crustal rocks. Compared to mantle peridotite, eclogites may have similar or higher seismic velocities but they have typically higher density^12,20–24^. Therefore, eclogitization of the lower crust may lead to the formation of a seismic Moho, which does not coincide with the petrological crust–mantle transition^10,25–28^: the seismic Moho is marked by a sharp increase in velocity at the top of an eclogitic lower crustal body, and the petrological crust–mantle boundary is marked by a change in composition from mafic to ultramafic rocks at the bottom of an eclogitic body, possibly without notable change in seismic velocity.

At high degree of eclogitization, the very high density below the seismic Moho, likely with density inversion at the crust–mantle boundary, may lead to delamination of the metamorphosed lower crust as proposed based on geological and geochemical arguments, and supported by numerical modelling^3,4,8,29–31^, but sparsely documented by geophysical evidence^9,32–34^, especially in cratonic settings. Delamination requires weak upper mantle rheology (e.g., temperatures >1500 °C^3^) and lower crustal temperature >900–1000 °C^31,35,36^, and the presence of decoupling zone may be important to initiate delamination^33,37^. If these conditions are not satisfied, thick lower crustal roots may be preserved, dependent on exact composition.

The presence of eclogites at around the Moho discontinuity has mostly been proposed based on geochemical data^3,10^ because their geophysical identification is difficult. Seismically imaged continental lower crust with fast Vp (7.2–7.6 km/s) usually does not extend over distances of >100–150 km^38^ and only a small number of seismic profiles have allowed for interpretation of eclogitic lower crust below the seismic Moho^39^. In cratonic settings, the presence of high-velocity, high-density material below the seismic Moho, indicative of an eclogitic body atop the mantle, has not yet been univocally documented, while the proposed eclogitic bodies atop the upper mantle of the Slave craton^27^, the west-central Finland (ca. 24° E, 62° N)^28^ and the Lapland Granulite Belt of the Fennoscandian Shield based on the non-reversed seismic profile^40^ do not extend laterally over >50–100 km. Furthermore, the origin of the only known exceptionally high Pn velocity (8.7 km/s) anomaly in the Siberian craton^41^, which may be caused by a high-velocity eclogitic body, is still uncertain^42,43^.

**Fig. 1:**
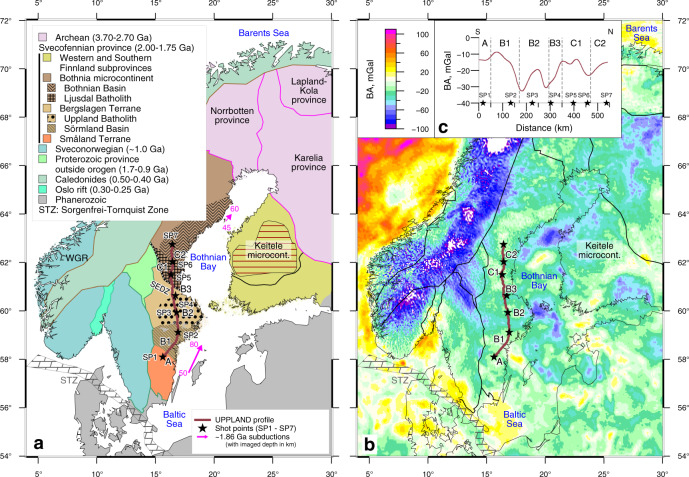
Tectonic sketch and Bouguer gravity anomaly maps with location of the UPPLAND seimic profile. (**a**) Tectonic sketch map, (**b**) Bouguer gravity anomaly map and (**c**) upward continued gravity data along the seismic profile. Black stars (SP1–SP7)—seismic source locations. Magenta lines—dip of Proterozoic subductions imaged seismically. Major tectonic units: A—Småland Terrane, B1—Sörmland Basin, B2—Uppland Batholith, B3—major deformation zone, C1—Ljusdal Batholith, C2—Bothnian Basin. SEDZ Störsjon–Edsbyn Deformation Zone, WGR Western Gneiss Region, STZ Sorgenfrei–Tornquist Zone.

Here we present a seismic velocity model for the upper 55–80 km of the central part of the Fennoscandian Shield along a newly acquired ca. 550-km-long seismic refraction/wide-angle reflection profile UPPLAND (Fig. 1), which we further detail by gravity modelling. The profile crosses four Paleoproterozoic terranes separated by major suture zones^44,45^, which allows for geophysical testing of the structure of the lower crust and the crust–mantle transition in paleosubduction settings. The profile is close to the central segment (profile distance km ca. 500–1100) of the FENNOLORA seismic profile^46–48^, which could not constrain the high seismic velocities in the lower crust. Therefore, the interpretations of the crustal lithology, the depth and nature of the seismic boundaries, and the nature of the crust–mantle transition in the Paleoproterozoic craton hitherto remain unconstrained. We document the presence of high-velocity, high-density material at the crust–mantle transition, which we interpret as the lowermost crust in eclogite facies preserved since the Paleoproterozoic (>1.8 Ga).

## Results

### Major lithospheric domains along the UPPLAND profile

From south to north, the seismic profile crosses the following major geological terranes in south-central Sweden (Fig. 1a):

A: Km 0–50: The Småland Terrane (1.83–1.79 Ga) is part of the Transscandinavian Igneous Belt (TIB, 1.85–1.67 Ga). The TIB intrusives, dominated by I- and A-type granitoids, are compositionally different from the subduction-related TTG (tonalite–trondhjemite–granodiorite) rocks of the Fennoscandian Shield and are interpreted to be emplaced at an active continental margin^49^. Possible traces of a fossil subduction zone are imaged offshore in the BABEL seismic profile B as strong NE-dipping mantle reflectors (dip angle 15–20°) at depths 50 to 80 km associated with a strong Pn-velocity contrast^50^ (Fig. 1a). A generally youngling age of intrusives southwards from the Småland terrane is explained by retreat of the northward subduction at 1.81–1.76 Ga^49^.

B: Km 50–350: The Bergslagen Terrane (Microcontinent) (1.90–1.75 Ga) of the SW Svecofennian orogen includes the Sörmland Basin and the Uppland Batholith, which is one of the major magmatic ore-bearing provinces in the Fennoscandian Shield. Anatectic 1.85–1.75 Ga granites near the TIB margin may be related to crustal thickening and major mafic underplating. The general absence of high-pressure metamorphic rocks, as in all of the Svecofennian orogen^44^, and the presence of low-pressure/high-temperature metamorphism is interpreted in favour of a continued subduction setting at 1.85–1.75 Ga^49^.

B1: Km 50–170: The Sörmland Basin dominated by metasedimentary migmatites is interpreted as the accretionary prism of 1.86 Ga northward subduction^51^.

B2: Km 170–295: The Uppland Batholith with a 1.90–1.87 Ga juvenile continental arc-derived crust was possibly formed above an continental-margin subduction zone^44^. 1.83–1.79 Ga felsic magmatism and coeval low-pressure/medium-temperature amphibolite facies metamorphism are attributed to crustal remelting in an extensional setting, such as continental back-arc.

B3: Km 320–350: A major 1.86–1.81 Ga deformation zone marks collision between the Bergslagen Terrane and the Central Svecofennian orogen^49^ and terminates at the Storsjön–Edsbyn Deformation Zone (SEDZ) at ca. km 350.

C. Km 350–550: The Bothnia Arc (Microcontinent) (1.86–1.80 Ga) comprises two major terranes separated by a wide arcuate network of 1.87–1.86 and 1.80 Ga subvertical shear zones (the Hassela Shear Zone centred at ca. km 480).

C1. Km 350–470: The Ljusdal Batholith, made of granitoids with minor amounts of mafic intrusions, is a relatively juvenile 1.85–1.84 Ga arc formed at an active continental margin. A major part of the batholith has been affected by low-P/high-T metamorphism and deformation at ~1.82 Ga^49^.

C2. Km 470–550: The Bothnian Basin may have formed above two subduction zones with opposite polarity. Abundant 1.86–1.80 Ga granitoids that form a 400 km long area of interconnected batholiths are covered by a ca. 10 km thick sequence of Paleoproterozoic, possibly marine, metasedimentary rocks^47^. Possible presence of Archaean crust is debated.

### Seismic data and interpretations

**Fig. 2:**
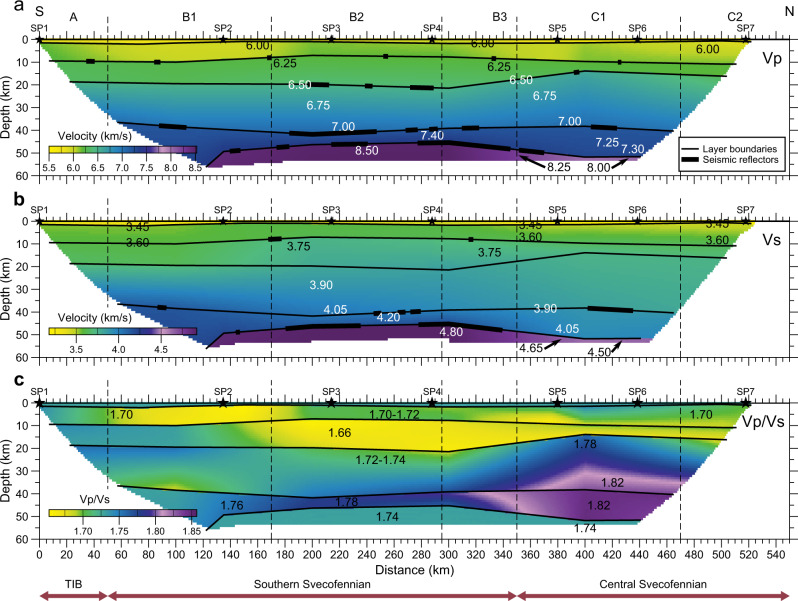
Seismic models along the profile. **a** P- and **b** S-wave velocity and **c** Vp/Vs ratio along the seismic profile. Seismic sources (SP1–SP7)—black stars; tectonic units as in Fig. 1. Velocity discontinuities—dashed lines; identified seismic reflections—thick black lines (shown in **a**, **b** only).

The new controlled-source seismic refraction/wide-angle reflection profile constrains in high resolution the velocity structure of the entire crust and the uppermost mantle down to ~55 km depth in the southwestern part of the Svecofennian orogen (Fig. 2). The profile was recorded by 593 short-period seismic recorders with an average station spacing of 950 m, reduced to ~800 m in the central 300 km of the profile and extended to about 1400 m towards both ends of the profile. The nominal distance between the 7 explosive sources ranges from 40 to 80 km (Fig. 1b). The largest charges with approximately 500 kg explosives were used at the two ends of the profile, and 360–475 kg of explosives were used in between (see ‘Methods’ and Supplementary Information).

The data were interpreted by traveltime picking of the main seismic phases from the crust and uppermost mantle and subsequent P- and S-wave raytracing traveltime modelling of the crustal and the uppermost mantle P- and S-velocity structures. Except for SP2 and SP5, where refracted Moho phases are ambiguous, the high-quality data allow for clear identification of crustal refractions (Pg) and reflections (PcP), and Moho refractions (Pn) and reflections (PmP). Besides, the northernmost shot, SP7, shows reflections after the Moho refracted arrivals from ~300 km offset (Supplementary Fig. 1).

Extensive testing of model resolution and uncertainty shows that the velocity structure of the crust and the uppermost mantle is well constrained by the newly acquired seismic data (‘Methods’ and Supplementary Information). The resolution of depth and velocity are ±1 km and ±0.1 km/s, respectively (Supplementary Fig. 2). Comparison of synthetic and observed gravity anomalies provides an additional constraint on the model.

### Seismic velocity model

The derived seismic velocity model (Fig. 2) challenges conventional models of the cratonic lower crust and shows the following characteristics:

1. Thick cratonic crust with the Moho depth at 50–52 km in the southern part of the profile and below the Bothnian Basin in the north and at 45–47 km in the central segment (the Uppland Batholith) where the lower crustal layer (Vp > 6.75 km/s) is also thin;
2. Thick (25–30 km) lower crustal layer (Vp > 6.8 km/s) below the Ljusdal Batholith where it makes 50–58% of the crust and thin lower crust below the Uppland Batholith (15–18 km, 33–38% of the crust) and below the northern Sörmland Basin (20–22 km, 40–44% of the crust), which is covered by ca. 8 km of metamorphosed felsic volcano-sedimentary layer;
3. Presence of a high-velocity (7.2–7.4 km/s) lowermost crustal layer along the entire ca. 350 km-long resolved profile section (from 120–150 to 480–500 km distance along the profile). This high-velocity layer was not identified in previous interpretations based on lower resolution seismic data^46,48^ but was just assumed^47^. This layer has a high Vp/Vs ratio (1.76–1.83) with maximum values below the Ljusdal Batholith (Fig. 2c);
4. Pronounced thinning of the high-velocity lowermost crustal layer from 10–14 km (22–26% of the crust) below the Ljusdal Batholith in the north to 5–7 km (10–15% of the crust) below the Uppland Batholith. Thus, crustal thinning below this area is dominantly related to thinning of the high-velocity basal crustal layer;
5. Sharp change in the average crustal Vp/Vs ratio across the SEDZ between the Southern and Central Svecofennian provinces, from 1.71 ± 0.06 below the Uppland Batholith to 1.78 ± 0.12 below the Ljusdal Batholith;
6. Exceptionally high Pn velocity (8.5–8.6 km/s) below the Bergslagen Terrane (with the highest values below the Uppland Batholith) over a minimum distance of 100–120 km. Across the SEDZ, the Pn velocity drops to normal values (8.0–8.1 km/s) below the Ljusdal Batholith. However, the upper mantle Vp/Vs ratio is normal (~1.74) along the entire profile;

The most intriguing features of the crustal and upper mantle structures along the profile include two well-resolved terranes, separated by the SEDZ, with distinct differences in physical properties:

(i) the Uppland Batholith of the SW Svecofennian province with a low average Vp/Vs crustal ratio (1.71) and a thin (5–7 km) but high-velocity (Vp ~ 7.4 km/s) lowermost crustal layer and an extremely fast uppermost mantle (Pn ~ 8.5–8.6 km/s); and
(ii) the Ljusdal Batholith of the Central Svecofennian province with a high average crustal Vp/Vs ratio (1.78) and anomalously thick lower crust (25–30 km) with thick high-velocity lowermost crust (10–14 km thick, Vp ~ 7.25 km/s), underlain by mantle with normal velocities (Pn ~ 8.0 km/s).

Despite significant variation in crustal structure, the average crustal velocity is constant (6.60–6.62 km/s) in the Uppland and the Ljusdal Batholiths due to relative variation in thickness and Vp in the upper and the lower crustal layers.

### Anomalous crustal and upper mantle structures

Two lithospheric features constrained by the new seismic data are highly unusual:

1. The presence of a continuous high-velocity (Vp > 7.3 km/s) lowermost crustal layer over a distance of at least 350 km is exceptional for Precambrian cratons (Figs. 2 and 3). In other cratons, a high-velocity lower crust has local character, ca. 100–150 km long or less^28,38,52–55^. The geodynamic origin of such high-velocity continental lower crustal layer is often attributed to magmatic underplating^38^, associated with paleocollisions (the Wyoming craton and the Keitele arc of the Western Finland subprovince of the Fennoscandian Shield)^52,53^ or lithosphere extension (the Donbas rift of the East European craton)^54^ or possibly hotspots (the Korosten anorthosite-rapakivi pluton in the Ukrainian Shield)^55^. Moreover, the Vp/Vs in the high-velocity cratonic lower crust, where determined^28,38^, does not show as high values (1.76–1.84) as along the UPPLAND profile (Fig. 3).

**Fig. 3:**
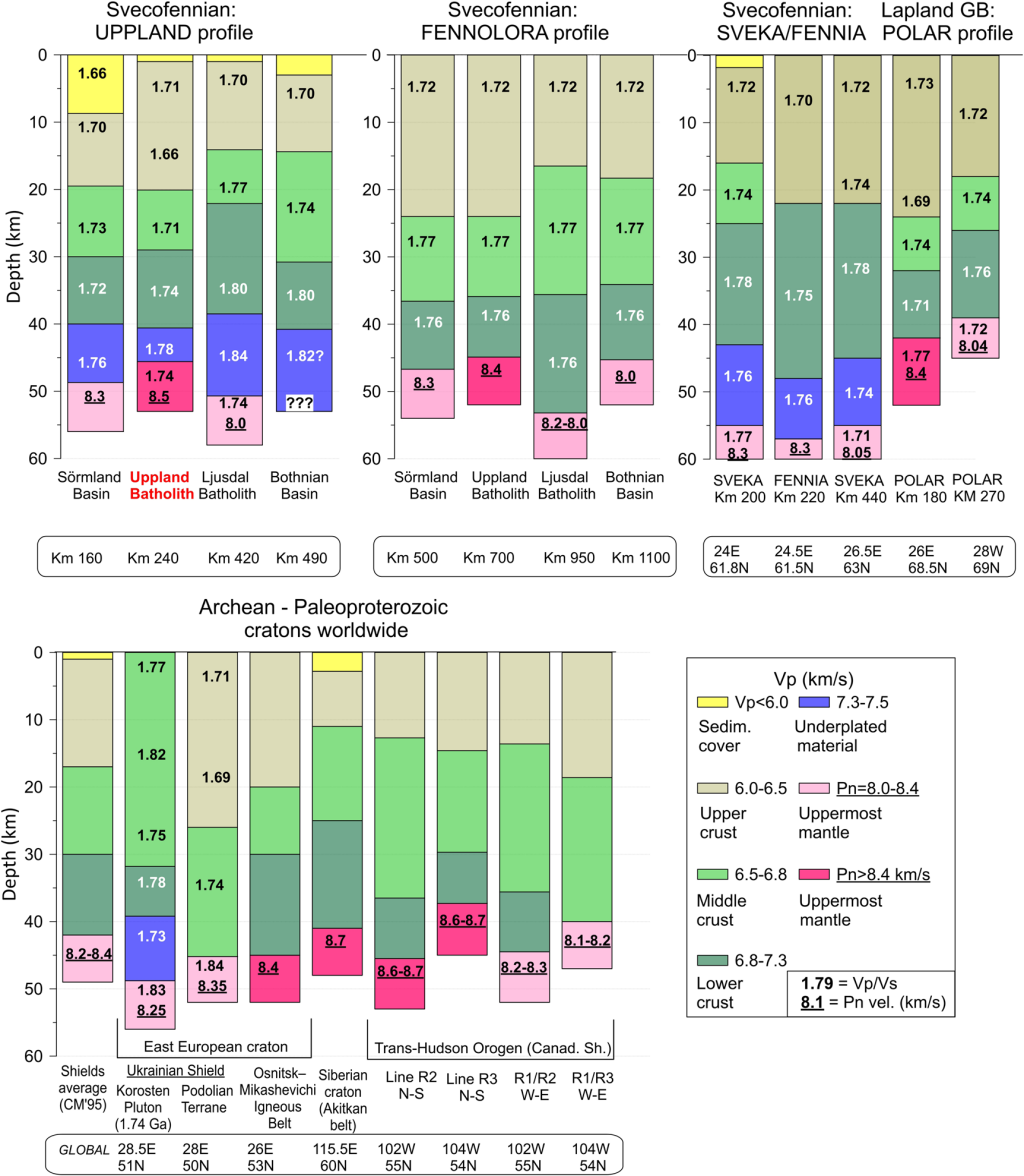
Vertical crustal seismic P-wave velocity sections. Sections for the Svecofennian province along the new UPPLAND seismic profile and the FENNOLORA profile (after ref. ^48^; Pn velocity from ref. ^47^) compared with sections for other cratonic regions with a high Pn velocity (>8.4 km/s): Baltic Shield^28,40^, East European Craton^55^, Siberian craton^41^, and Trans-Hudson orogen of the Canadian Shield. Global average for shields and platforms^11^ (CM’95) is shown for comparison. Numbers—Vp/Vs ratio, where determined; underlined numbers—Pn velocity (km/s). Text in boxes—profile distance for the two Svecofennian profiles and coordinates for other cratons.
2. Extremely high Pn velocities (8.5–8.6 km/s) below the Uppland Batholith are among the highest reported globally. The highest known Pn-velocity of 8.7 km/s below the Markha kimberlite province (around pipe Mirnyi) of the Siberian craton^41^ is observed on two perpendicular profiles, which therefore excludes anisotropy as a cause of the extreme uppermost mantle velocities. Nevertheless, strong anisotropy of mantle peridotites instead of the presence of abundant high-Vp eclogites was proposed as explanation for the unusually high Pn velocity based on studies of a small set of mantle-derived xenoliths from this and other kimberlite fields in Siberia, which nonetheless report eclogitic Vp values, similar to seismic observations^42,43^. High Pn velocity (8.6–8.7 km/s) is also observed in the Trans-Hudson Orogen of the Canadian Shield, but only along ca. 100 km long sub-longitudinal segments, where a crossing profile has normal (8.1–8.3 km/s) Pn velocity, such that mantle anisotropy is an obvious explanation of the observations^56^ (Fig. 3). Alternatively, the high Pn may be explained by the presence of a relic eclogitic slab in the uppermost mantle associated with Paleoproterozoic oceanic subduction^57^.

In the absence of E-W striking high-quality seismic profiles across the Bergslagen Terrane, mantle anisotropy as the cause of the high uppermost mantle Vp beneath the central segment of the profile cannot be ruled out. However, the Uppland Batholith is unique with the combination of: (i) high-velocity (Vp > 7.3 km/s) lowermost crust, which is (ii) ca. 6–8 km thinner than the adjacent terranes, and (iii) is situated immediately above extremely high velocity (Vp ~ 8.5–8.6 km/s) uppermost mantle. In contrast, the Siberian craton in the area with the extreme Pn velocity has relatively small lower crustal velocities (6.8–6.9 km/s)^41^, while a section of the Wyoming craton with fast lower crust (Vp > 7.5 km/s) has normal uppermost mantle velocities (Pn = 8.05–8.2 km/s)^52^, similar to the Ljusdal Batholith. We interpret the anomalous velocity structure below the Uppland Batholith by eclogitization of most of the original lower crust.

### Interpretation of lithology

**Fig. 4:**
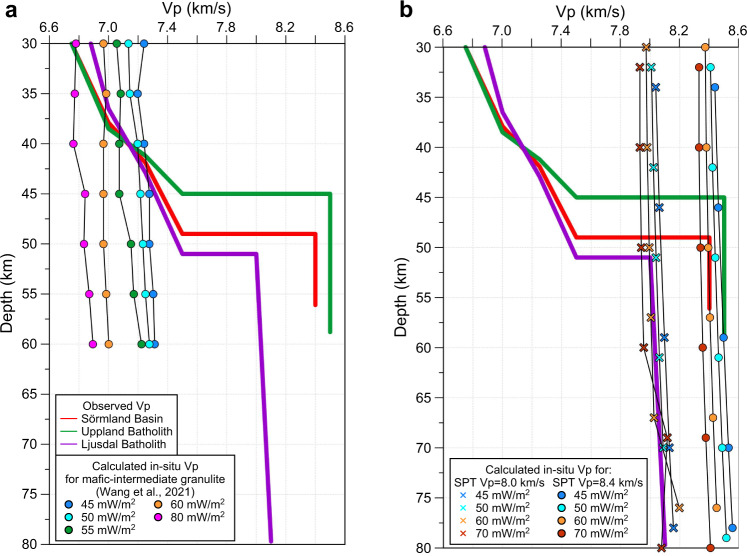
Comparison of observed Vp with in situ Vp calculated for major relevant rock types. Solid colour lines—observed vertical Vp profiles at three locations (170, 240 and 420 km distance along the profile). Thin lines—Vp calculated **a** for mafic-intermediate granulite composition of the lower crust^9^ by the thermodynamic code Perple_X^58^ and **b** for uppermost mantle with STP (at room (standard) pressure and temperature) Vp = 8.0 and 8.4 km/s using pressure and temperature Vp derivatives of 0.2 km/s/GPa and −4 × 10^−4^ km/s/K^6,20^. Colour-coded symbols correspond to various heat flow, which controls lithosphere geotherms (Fig. 5).

**Fig. 5:**
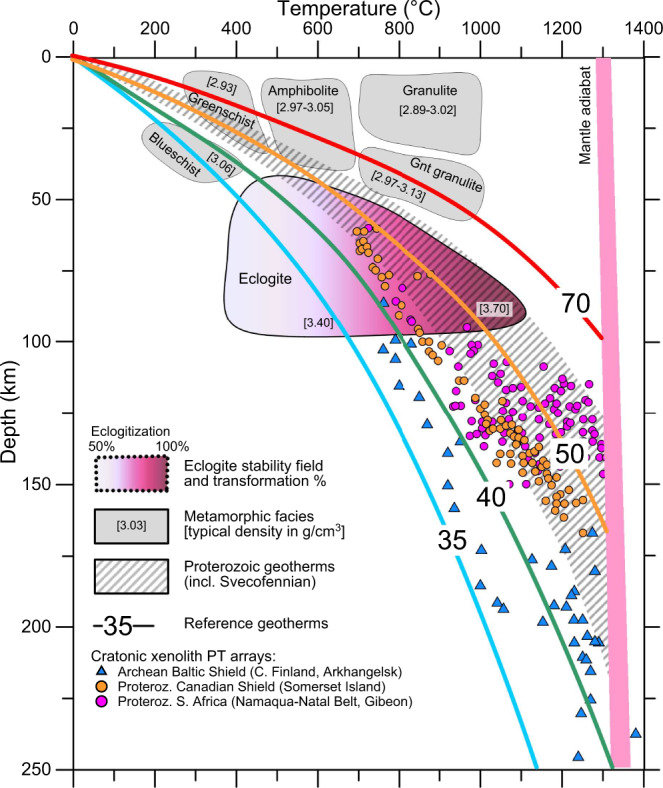
Continental geotherms based on heat flow^70^. Labels at colour lines—surface heat flow. Hatched region—geotherms in the Svecofennian province. Colour symbols—xenolith P–T arrays (c.f. ^70^) from Archaean terranes of Fennoscandia (triangles) and Proterozoic terranes of the Canadian Shield and the Kalahari craton (circles); no xenolith P–T arrays are available for the Svecofennian province. Grey shading—metamorphic facies pertinent to eclogite formation^15^; pink shading—degree of eclogitization^15^. Numbers in brackets—typical densities.

To interpret the composition of the anomalous lower crust and uppermost mantle along the new seismic profile, we compare upper lithospheric Vp-depth profiles with Vp-depth profiles calculated by the thermodynamic code Perple_X^58^ for typical lower crustal lithologies^9^ and for in situ upper mantle at relevant pressures and temperatures (Fig. 4). Regional lithosphere geotherms^59^ are consistent with xenolith P–T arrays from other Proterozoic terranes and follow reference geotherms from 45 to 55–60 mW/m^2^, which cross the eclogite stability field at depths >45 km (Fig. 5), which corresponds to the depth of the seismic crust–mantle transition below the Bergslagen Terrane (Fig. 2). The interpreted Vp velocities in the lower crust are consistent with granulites of mafic to intermediate compositions (Fig. 4a), while the observed uppermost mantle velocities below the Bergslagen Terrane (8.5–8.6 km/s) and the Ljusdal Batholith are consistent with STP Pn-wave velocities of ca. 8.4 and 8.0 km/s, correspondingly (Fig. 4b).

**Fig. 6:**
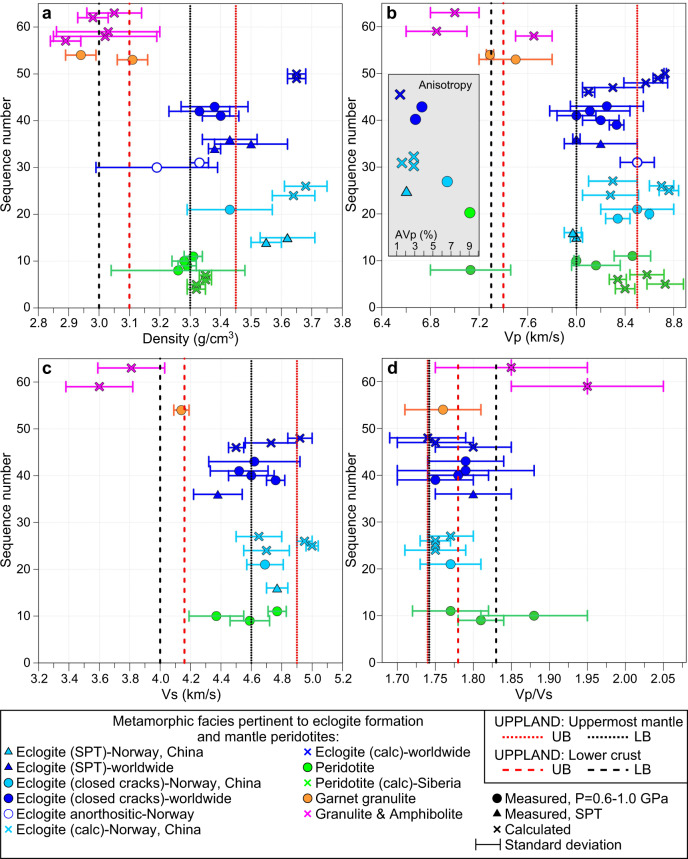
Plots of observed and calculated physical parameters for various rock types (based on Artemieva, 2020, unpublished compilation; for references, see Supplementary Material). **a** Density, **b**, **c** seismic P- and S-wave velocities and **d** Vp/Vs ratio in peridotite (green), eclogites (light blue—UHP terranes of Norway and China; dark blue—other locations), and metamorphic facies pertinent to eclogite formation (orange and magenta). Laboratory measurements are at room (STP) conditions (triangles) and at room temperature and *P* = 0.6–1.0 GPa sufficient to close microcracks (circles); calculated values are shown by crosses. Inset in **b**: Vp anisotropy in peridotites and eclogites. Vertical lines—our results for the Uppland (red) and Ljusdal (black) Batholiths (lower crust—dashed lines, uppermost mantle—dotted lines).

Laboratory measurements at *P* = 0.6–1.0 GPa (i.e., with closed microcracks) and calculations based on electron back-scattered detection show that in peridotites and eclogites seismic Vp and Vs show some overlap, with typical Vp ranging from 8.2 to 8.6–8.7 km/s in both rock types^12,21,23,24,42,43,60^, although peridotites of the Japan island arc have a very low average Vp = 7.13 ± 0.33 km/s^12^ and values >8.3–8.4 km/s in peridotite were measured in three Siberian xenoliths only^42^. Otherwise such high values are only based on calculated average velocities for assumed mineral assemblages^23^ (Fig. 6). Our compilation indicates that eclogites may statistically have a slightly higher Vs (typically >4.6 km/s) and slightly lower Vp/Vs than peridotites, especially for samples from the ultra-high pressure (UHP) terranes of Norway and China (typical Vp/Vs = 1.74–1.78 as compared to >1.77 in peridotites; Fig. 6).

Densities of eclogites and peridotites are significantly different: 3.23–3.39 g/cm^3^ for peridotites with mean values of 3.31–3.35 g/cm^3^ and up to 3.75 g/cm^3^ for eclogites^9^, with common values of 3.45–3.50 g/cm^3^ reported worldwide^6,15,24^, and as high as ca. 3.55–3.70 g/cm^3^ in UHP eclogites from Norway and China^16,20,60^ (Fig. 6a). Laboratory-measured eclogite densities are essentially controlled by the composition of rocks undergoing eclogitization and the metamorphic grade^19^ and demonstrate a significant density contrast (0.2–0.3 g/cm^3^) between mantle peridotites and eclogites, which may allow for discrimination by gravity interpretation if the eclogite bodies have sufficient volume.

**Fig. 7:**
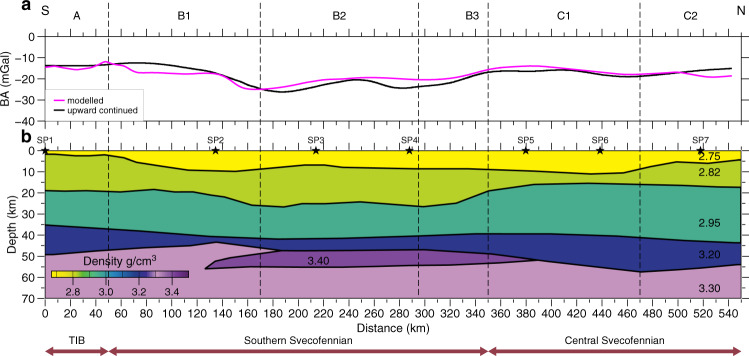
Gravity model along the UPPLAND seismic profile. Calculated gravity compared to EGM2008 gravity data^68^ **a** for the density model **b** based on the velocity model (Fig. 2). Gravity data are 50 km upward continued to filter short-wavelength shallow structures. The correlation between the calculated and observed data supports interpretations of the velocity model.

Our 2.5D gravity model (Fig. 7) is initially constrained by converting the velocity model into densities (‘Methods’ and Supplementary Material). It includes a normal cratonic crust with lower crustal density of 2.95–3.1 g/cm^3^ underlain by a 6–8 km thick high-density (3.45 g/cm^3^) body below the Uppland Batholith of the Southern Svecofennian orogen, whereas the upper mantle of the Ljusdal Batholith of the Central Svecofennian province has normal density of 3.30 g/cm^3^ (Fig. 7). The calculated gravity response corresponds to the observed gravity data with 100 km wavelength gravity high in the centre of the profile within a wider low-amplitude gravity low. Gravity modelling allows for testing the likely depth extent of the anomalous high-Vp, high-density body below the seismic Moho, which cannot be resolved by the seismic model. The base of the high-density body is located at a depth comparable to the seismic Moho depth in the surrounding parts of the profile, which suggests that the high-density body consists of original lower crustal material, which has been metamorphosed into eclogite facies.

## Discussion

The unusual structure of the lower crust and the uppermost mantle below the Uppland Batholith of the Bergslagen Terrane is best explained by eclogitization of the lowermost section of the original lower crust (Fig. 6), which seismically now is part of the mantle. Therefore, the seismic Moho below the Paleoproterozoic Bergslagen Terrane is not a compositional but a metamorphic boundary caused by the transition from granulite to eclogite facies, where the transformation reached a high degree of eclogitization. This interpretation is supported by the strong reduction in the lower crustal thickness, the presence of the unusually fast but thin lowermost crustal layer, and the extremely high-Vp, low Vp/Vs and high-density uppermost mantle below the Uppland Batholith.

**Fig. 8:**
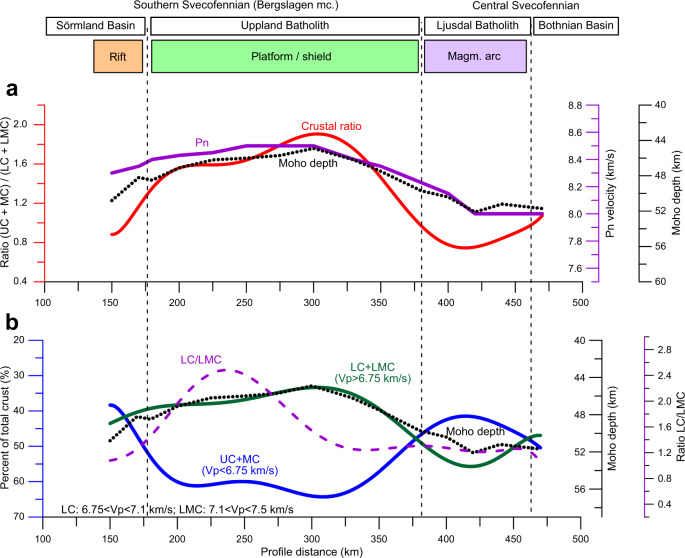
Variations in the Moho depth, Pn velocity and thickness of the crustal layers along the profile. **a** Ratio between thickness of the felsic-intermediate crustal layer (Vp < 6.75 km/s) to the mafic lower crustal layer (6.75 < Vp < 7.5 km/s) (red), together with Pn velocity (purple) and Moho-depth (stippled black); please notice the close coincidence. **b** Moho depth (black), relative thicknesses (in %) of the felsic-intermediate crustal layer (UC + MC, Vp < 6.75 km/s) (blue) and of the mafic lower crustal layer (LC + LMC, 6.75 < Vp < 7.5 km/s) (green) to total crustal thickness; also shown is the ratio LC/LMC of the thicknesses of the ‘normal’ lower crust (LC, 6.75 < Vp < 7.1 km/s) to the high-velocity lowermost crust (LMC, 7.1 < Vp < 7.5 km/s) (dashed purple line). The tectonic interpretation on the top is based on Fig. 9.

We observe striking correlations (*R*^2^ = 0.86–0.90) between Moho depth, uppermost mantle Pn velocity, thickness of the lower crust (Vp > 6.75 km/s), and ratio of the thicknesses of the upper-middle (Vp < 6.75 km/s) to mafic lower crust along the entire resolved part of the seismic profile (Fig. 8 and Supplementary Fig. 7). These correlations indicate a generic link between modification of the crust and the anomalously high sub-Moho mantle velocities.

**Fig. 9:**
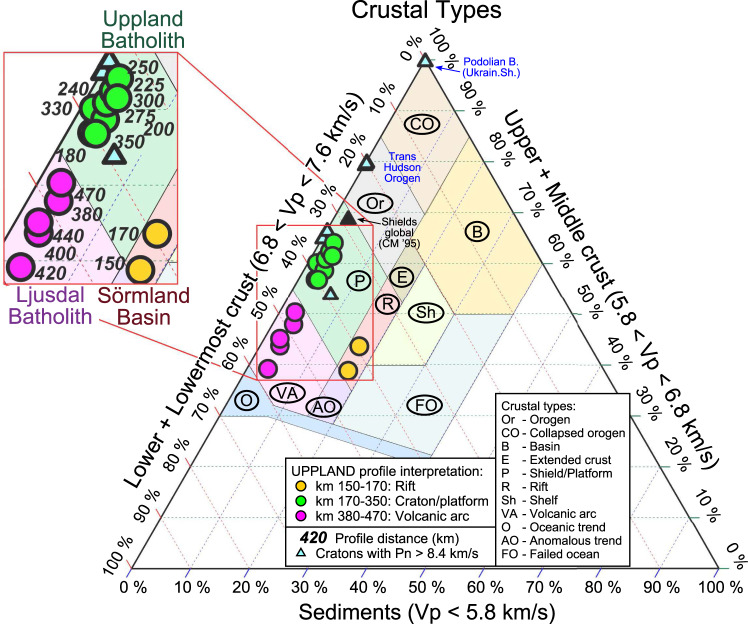
Ternary plot of the fractions of the metasedimentary, felsic-intermediate and mafic crustal layers in the total crustal column. Coloured circles—crustal structure based on our seismic model (Fig. 2) sampled along the profile (orange—Sörmland Basin, green—Uppland Batholith, magenta—Ljusdal Batholith; profile distance in km is marked in the zoomed insert). Blue triangles—cratons globally with Pn > 8.4 km/s (Fig. 3); black triangle—global average for shields and platforms^11^. Background colours with text labels mark typical tectonic domains identified in a global analysis^61^.

The ratio of the thicknesses of the three major crustal layers (sediments, upper–middle crust, and lower crust), as illustrated by ternary diagrams (Fig. 9), is an indicator of the geodynamic origin of the crust^61^. Cratons with high Pn velocity (Fig. 3) plot into the shield/platform domain (Fig. 9), except for the Trans-Hudson Orogen, which plots as a typical orogen, and for the Paleoproterozoic Korosten pluton of the Ukrainian Shield (Fig. 3), which plots within the domain of collapsed orogenic crust.

The crustal structure of the Paleoproterozoic cratonic terranes, crossed by the UPPLAND seismic profile, falls into three clearly distinct clusters. They include: (1) an extensional crust typical of continental rifts below the Sörmland Basin, (2) a typical shield/platform crust below the Uppland Batholith of the Southern Svecofennian orogen, and (3) a volcanic (magmatic) arc crust below the Ljusdal Batholith of the Central Svecofennian orogen, possibly extending below the southern margin of the Bothnian Basin at profile distance km 470, where the model loses resolution for the Moho depth and the lower crust (Fig. 9).

1. An extensional paleo-regime of the Sörmland Basin in southern Bergslagen is supported by the presence of low (5.5–5.8 km/s) seismic velocities down to ca. 8 km depth (Fig. 2) associated with metasediments. The basin may have formed by lithosphere extension in a forearc setting, possibly later overprinted by a backarc extensional regime, which caused low-P/high-T amphibolite-to-granulite facies metamorphism at ca. 1.85–1.79 Ga and voluminous mafic underplating that produced a thick crust with a ca. 10 km-thick high-velocity lower crustal layer.
2. The juvenile 1.90–1.87 Ga arc crust of the Uppland Batholith was formed at an active continental margin above the northward subduction. In case the original continental arc crust was preserved, it would have plotted on the ternary diagram within the same cluster as the Ljusdal Batholith (Fig. 9), which requires additional 9–13 km of mafic crustal material, assuming that the present-day thickness of the upper-to-middle crust is preserved since the Paleoproterozoic. It implies that the present-day petrological Moho beneath the batholith is at a 54–58 km depth, consistent with the gravity model (Fig. 7), which is deeper than the maximal depth resolved seismically (Fig. 2). The sharp change in Vp velocity at ca. 45 km depth, interpreted as the seismic Moho, corresponds to the top of the eclogitic layer, which chemically is part of the lower cratonic crust. Laboratory studies of exposed metamorphic rocks in SW Norway have demonstrated that the Moho may be relatively sharp where a mafic lower crust in granulite facies is in direct contact with eclogite^19,62^.
  The significant amount of water required for the formation of abundant eclogite-facies rocks over geological time^13^ may have been delivered by subduction of oceanic lithosphere beneath the Bergslagen magmatic arc^18,63^, as imaged offshore at 50 to 80 km depth^50^ (Fig. 1a). Based on Pn velocity and the uppermost mantle density required by gravity data, we estimate that 50–70% of mafic lower crustal material has transformed into eclogite facies below the seismic Moho (the value depends on the choice of parameters for peridotite and eclogite, Fig. 6).
  Under certain thermo-chemical conditions, thick and strong high-grade crustal roots can be preserved since the Precambrian^35,37^. Although lower mafic crust converted to eclogite may delaminate and sink into the mantle^3,8,29,36^, the presence of a very high Pn velocity in very dense uppermost mantle material indicates that the eclogitic body is still present beneath the Uppland Batholith. This conclusion is consistent with a cold regional geotherm and a Moho temperature of 500–700 °C (Fig. 5), since delamination of a 5–30 km thick eclogitic lower crustal layer with density of ca. 3.4–3.6 g/cm^3^ requires a lower crustal temperature of >900–1000 °C^30,35,36^. Regional studies suggest a generic link between eclogite bodies in the lower crust and lithosphere deformation^39^. We speculate that the dynamics of the major deformation zone, SEDZ, at the boundary between the Bergslagen Terrane and the Ljusdal Batholith may significantly be controlled by the inferred eclogitic body below the Uppland Batholith.
3. Our results document two principally different evolutionary models for Precambrian subduction, primarily controlled by the fluid and temperature regime in the slab, which in turn is controlled by the nature of subducting plate (oceanic or continental), as well as by lithosphere structure of the upper plate. Our results indicate that the Ljusdal Batholith of the Central Svecofennian orogen, formed at an active continental margin during Paleoproterozoic collision of the Bergslagen and the Bothnian Basin arc, has preserved its thick lower crustal layer beneath a thinned middle crust (Fig. 8). Its seismic velocity and density structures are consistent with magmatic underplating and presence of mafic rocks in the crust, which also explains the peak in the low-P/high-T metamorphism at ca. 1.82–1.80 Ga^49,53^. Normal uppermost mantle velocity and density suggest that the seismic Moho marks the top of the peridotite mantle. We infer that either Paleoproterozoic subduction below the Ljusdal Batholith did not involve an oceanic plate, or that temperature and fluid regime did not favour the granulite-to-eclogite facies transformation as in the Uppland Batholith. Our model contrasts one of geodynamic interpretations of the Central Svecofennian orogen in Finland, where an assumed lack of density contrast across the present Moho in a region with a high-velocity lower crust (Vp ~ 7.3–7.5 km/s at depths of 40–63 km) is related to Paleoproterozoic delamination of eclogitic lower crust^36^. Instead, delamination should rather create a strong density contrast between the crust and uppermost mantle. Additionally, our extensive crustal database shows that the region with a high-velocity lower crust in central Finland may be very local^64^. Gravity and seismic data for the Ljusdal Batholith (Figs. 2 and 7) imply a different geodynamic scenario for the Central Svecofennian orogen in Sweden than for the Central Svecofennian orogen in Finland^36^, and the genetic connection between the two regions of the Central Svecofennian orogen is still a matter of controversy.

A global analysis of wide-angle seismic profiles across passive margins shows that the seismic Moho marks the top of the mantle in 94% of seismic data^39^. In contrast, the seismic and petrological crust–mantle transitions do not coincide below the Uppland Batholith of the Southern Svecofennian orogen. Our finding of a ca. 150–200 km long and 6–8 km thick eclogite body atop the petrological mantle in a Paleoproterozoic craton is unusual and presents the first well-documented geophysical evidence for the presence of a large eclogitic body at the cratonic crust–mantle transition. The kimberlite province of the Siberian craton is, so far, the only other cratonic region where possible extensive presence of eclogites in the uppermost mantle is suggested by seismic data^41^, but debated on various grounds^42,43^, while in the Finnish part of the Svecofennian orogen the possible presence of a 50–100 km long eclogitic mantle is local^40^.

Our results show that large continuous sections of eclogitized lower crust may remain gravitationally stable for nearly 2 billion years. Therefore, our finding of a highly unusual structure of the Paleoproterozoic Bergslagen continental arc opens new perspectives for understanding Precambrian geodynamics, the nature of the crust–mantle transition, long-term stability of Precambrian cratons, models of the crustal growth and recycling through time and geochemical budgets for the Earth’s secular evolution.

## Methods

### Seismic data

Our approximately 550 km-long refraction/wide-angle reflection profile was acquired in May–June 2017 in the central part of the Baltic Shield in Sweden between 58° N and 62.8° N latitude along ~16.6° E longitude and slightly further west in the southernmost 100 km (Fig. 1). A mix of 593 1 C and 3 C seismic recorders with a mean spacing of 950 m recorded 7 shots 40–80 km apart located along the profile. The 1 C seismic recorders were connected to 4.5 Hz spike-type geophones, and micro-electromechanical system-based 3 C digital sensors were used for the 3 C recorders. The sampling rate ranges between 1 and 4 ms for different seismic recorders (Supplementary Table 1). All data were resampled to 5 ms before processing and modelling. The charge sizes of the 7 shots range between 360 and 500 kg, with the largest charges positioned at the two ends of the profile (shots SP1 and SP7). All charges were divided into several boreholes at a depth of 30 and 11 m for SP4 (Supplementary Table 2).

### Seismic data analysis

As a first step, phase correlation and traveltime picking of P- and S-wave phases were conducted followed by building the crustal velocity structures by raytracing traveltime modelling. We used the szplot software package^65^ for correlation and traveltime picking. The raytracing was performed using the rayinvr software^65^ together with the graphical user interface Pray^66^. Different features of the picked traveltimes of reflected and refracted phases were included in the modelling of the velocities in the crust and uppermost mantle. We matched the apparent velocities of refracted phases and checked for matches of reciprocal arrivals to derive the velocity models. Reversed arrivals constrain the model well.

### Resolution of the seismic models

We use the diagonal values of the resolution matrix to assess the depth and velocity resolution of the models^67^. In general, model parameters are reasonably well resolved for diagonal values >0.5. We tested the resolution by adding perturbations of ±1 and ±2 km to the depth nodes of the final model for the P-wave model and depth perturbations of ±3 km for the S-wave model. We then calculated the diagonal values of the resolution matrix by raytracing inversion for the crustal velocity structure. Essentially, nearly all nodes are resolvable within 1 km at all depths (Supplementary Fig. 2a, c), and a few nodes are only resolved by 2 or 3 km. Only the third interface of the S-wave model is not well resolved. The velocity resolution was checked for perturbations of ±0.1 and ±0.2 km/s. (Supplementary Fig. 2b, d). The nodes are all well resolved for 0.1 km/s perturbation, also below the third interface in the S-wave model. Overall, the resolution of depth and velocity is ±1 km and 0.1 km/s, respectively.

### Traveltime fit for seismic phases

In general, the shot gathers have good quality of traceable P- and S-wave phases with clear onsets, which allows a precise determination of the arrival times (Supplementary Figs. 3 and 4). We trace the P-wave arrivals in the crust (Pg) to offsets beyond 200 km in all the seismic sections and the S-waves (Sg) to offsets beyond 140 km. Reciprocity was checked for the signals in all sections. The refracted, first-arrival phases have high signal-to-noise ratio, and also the onset of intra-crustal reflections can be reliably picked.

We divide the refracted P-wave arrivals in the crust into five different parts (Pg1, Pg2, Pg3, Pg4, Pg5) plus the refracted P-wave below the Moho (Pn). P-wave reflections are observed from three intra-crustal interfaces (Pc2P, Pc3P, Pc4P) and the Moho. The velocities in the crust in the central part of the profile are constrained by several reversed refracted Pg-arrivals and confirmed by reflection phases.

- The Pg1 phase in the uppermost crust is visible in all shot gathers to offsets of up to 25 km. At SP4, the first segment of the Pg1 phase is not recorded since the shot location was around 15 km off the profile. The dips of Pg1 represent the apparent velocities above the first interface C1. The slopes range between 5.7 and 6.0 km/s. No reflection was detectable in the seismic sections.
- The Pg2 phase, identified in all seismic sections, has a higher apparent velocity than Pg1 ranging between 6.0 and 6.2 km/s and can be traced between 100 and 145 km offset. The reflection phase Pc2P from interface C2 at depths of ~10 km is observable by a few picks close to the Pg2 phases in SP1, SP2, SP3, SP4 and SP5 but not in SP6 and SP7.
- The Pg3 phase is not observable in the shot gathers of the two northernmost shots, SP6 and SP7, since the third layer is relatively thin with a thickness of 4.5 km and the first arrivals could not clearly be distinguished from the phase Pg4. In the other seismic sections is the Pg3 phase identified up to offsets of ~200 km with velocities between 6.2 and 6.4 km/s. Reflections from the C3 interface at depths of ~14–21 km are detectable in the seismic sections of SP2, SP3, SP4, SP5 and SP6 and help to constrain the velocity.
- The Pg4 phase is clearly identifiable in all shot gathers up to 230 km offset and the slope has velocities between 6.5 and 7.0 km/s. The interface C4 at 35–42 km depth is also reflecting phases (Pc4P) for all shots and is traced over longer offset intervals than the reflection phases from interfaces above.
- The Pg5 phase is just observable in seismic sections of SP1, SP2 and SP5 to offsets between 270 and 280 km. The velocities are around 7.2 km/s.
- The correlatable PmP phases in the shot gathers of shots SP1, SP3, SP4, SP5 and SP6 show different waveform and amplitude. The PmP phase for shot SP1 is traced from offset 200 to 230 km. In other parts of the section, it was not clearly pickable. However, for the more central shot points SP3 and SP4 the PmP reflection is identified over larger offset intervals ranging from 100 to 260 km, whereas the northern shot points SP5 and SP6 have a smaller offset interval from 145 to 215 km.
- The Pn phases are correlated in the northern and southern shots (SP1, SP3, SP6, SP7), with the clearest arrivals in the seismic sections of shots from the ends of the profile (SP1 and SP7). For SP1, the Pn phase is identified from 230 to 340 km offset interval and for SP7 from 230 to 300 km offset. The seismic sections of SP3 and SP6 have smaller offset intervals for the Pn phase with 200–280 km for SP3 and 210–250 km for SP6, but with three clear picks at far offset in SP6 at 335, 340 and 350 km. All in all, the Pn phases have good ray coverage in the uppermost mantle between 120 and 360 km distance along the profile.
- The slope of Pn phase is around 8.5 km/s. To verify this high velocity, different velocities in the upper mantle were tested (Supplementary Fig. 6). The upper red line shows the calculated arrivals with velocity of 8.4 km/s in the upper mantle of B2 and the bottom line for 8.6 km/s. The area in between is marked blue and shows that the velocity in the upper mantle is within that range.

We also trace S-wave phases, although the signal-to-noise ratio is lower than for the P-wave. As for the P-wave phases, we divide the refracted S-wave phase in the crust (Sg) into five parts (Sg1, Sg2, Sg3, Sg4, Sg5). Furthermore, we observe the refracted S-wave below the Moho (Sn) and the S-wave reflection of the Moho (SmS). Intra-crustal S-wave reflections are detectable from the second (C2) and fourth (C4) interface. Overall, the S-wave velocity model has a lower ray coverage than the P-wave velocity model, but it is constrained in its central parts.

- The first three Sg phases (Sg1, Sg2, Sg3) are correlated in all seismic sections. The Sg1 phase in the uppermost crust ranges is observed at offsets from 7 to 17 km with an apparent velocity of 3.1–3.2 km/s. The Sg2 phase is confidently picked to offsets of 60 km with apparent velocities of 3.5–3.6 km/s. At offsets from 60 to 100 km, we identify reflections from the second interface C2 (Sc2S) in the seismic sections of SP2, SP3 and SP4 in 10 km-long offset ranges between 60 and 70 km. Sg3 ranges between 100 and 200 km with apparent velocities of about 3.7 km/s. At large offsets, the Sg phases loses energy.
- The Sg4 phase is traced in the shot gathers of shot SP1, SP4, SP6 and SP7 in offset ranges from 160 to 240 km, and only 4 picks are made for the Sg5 phase from offsets of 260 to 270 km for SP1. The Sc4S reflection phase is correlated in the sections for SP1, SP2 and SP7 in the offset interval of 150–217 km and in far offset from 210 to 290 km in SP2.
- The amplitude of the reflection from the Moho (SmS) is relatively large, and the SmS phase is identified in all seismic sections, except for SP7, at offsets from 100 to 230 km. In particular, SmS is observed over large distances of up to 110 km for the three central shots (SP3, SP4, SP5).
- The refracted S-wave below the Moho (Sn) is observable in SP1, SP3 and SP7 from 250 to 280 km offset. The clearest arrival of Sn is in SP7.

The resulting raytracing velocity models for P- and S-waves are constrained by around 94% of the picked traveltimes (Supplementary Tables 3 and 4). We estimate the uncertainty of the picked traveltimes to 100 ms for the Pg phases and 150 ms for the intra-crustal reflections, PmP and Pn. For the S-wave phases, we estimate the uncertainties to 150 ms for the Sg phases and 200 ms for the other S-wave phases (ScS, SmS, Sn). The overall *χ*^2^ = 0.3 for phases constraining P-wave velocity model and 0.7 for the phases constraining the S-wave velocity model. *χ*^2^ would be 1 if the root-mean-square (RMS) traveltime residual would be the same as the estimated uncertainty. It is <1 if the RMS is smaller than the estimated uncertainty. The average RMS of our models is with 66 ms for the P-wave and 140 ms for the S-wave smaller than the estimated picking error. Altogether, we obtain a good fit of the calculated traveltimes with the picked traveltimes with only a few outliers. The ray coverage (Supplementary Fig. 5) and residuals of the traveltimes (Supplementary Tables 3 and 4) provide a good indication on how well the velocity model is constrained by the interpreted seismic phases. The P-wave velocity model is slightly better constrained than the S-wave velocity model due to the higher accuracy picks and better ray coverage.

### Gravity test

The P-wave velocity model was converted to a two-dimensional density section and the calculated gravity response was compared with the EGM2008 gravity data^68^ in order to evaluate the robustness of the velocity model and to assess the reliability of the interpretation of an eclogite body in the lower crust. We used the velocity–density relation^69^ as a reasonable model for continental crystalline rock settings.

To focus on deep features, the gravity data were upward continued to 50 km and used for calculating the gravity response of the converted 2.5D model, which is 600 km long with 300 km lateral extension in each direction. The model includes nine layers with densities ranging from 2.72 g/cm^3^ in upper crust to 3.30 g/cm^3^ in the upper mantle at depths from ca. 48 to 100 km. A 6–8 km-thick, high-density body immediately below the seismic Moho represents the eclogitic material at a depth range of 45–55 km. Tests show that the forward response of the model matches the upward continued observed gravity data. We conclude that the presence of an eclogite body is consistent with and is also supported by the gravity data.

## Supplementary information

Supplementary Information

## Data Availability

All data are available in Supplementary Figs. 3 and 4.

## Code availability

All applied computer codes are available from the authors’ website. The raytracing code from Colin Zelt is available at: https://terra.rice.edu/department/faculty/zelt/rayinvr.html. The GUI from Tanja Fromm is available at: https://sourceforge.net/projects/pray-plot-rayinvr/. The thermodynamic code Perple_X from James Connolly is available at: https://www.perplex.ethz.ch/.
