# Supplementary material for: Long-lived Paleoproterozoic eclogitic lower crust
Source: Nat Commun. 2021 Nov 12;12:6553. doi: 10.1038/s41467-021-26878-5 (PMC8589968; doi:10.1038/s41467-021-26878-5)
Supplement: Supplementary file 1 — Supplementary Information [file 41467_2021_26878_MOESM1_ESM.pdf]

Supplementary Information for

Long-lived Paleoproterozoic eclogitic lower crust

Sebastian Buntin, Irina M. Artemieva, Alireza Malehmir, Hans Thybo, Michal Malinowski, Karin Högdahl, Tomasz Janik and Stefan Buske

Correspondence to: sebastian.buntin@geo.uu.se

This PDF file includes:

Supplementary Figure 1. **Ray coverage and traveltime fit for SP1 and SP7.**
Supplementary Figure 2. **Resolution test for the P- and S-wave velocity model.**
Supplementary Figure 3. **Seismic sections for the seven shots (P-wave).**
Supplementary Figure 4. **Seismic sections for the seven shots (S-wave).**
Supplementary Figure 5. **Ray tracing results for different seismic phases.**
Supplementary Figure 6. **Test of upper mantle velocity.**
Supplementary Figure 7. **X-Y plots of all parameters.**
Supplementary Table 1. **Seismic station parameters for data acquisition.**
Supplementary Table 2. **Shot parameters for data acquisition.**
Supplementary Table 3. **Statistical parameters of seismic P-wave modelling for different picks.**
Supplementary Table 4. **Statistical parameters of seismic S-wave modelling for different picks.**
Supplementary References.

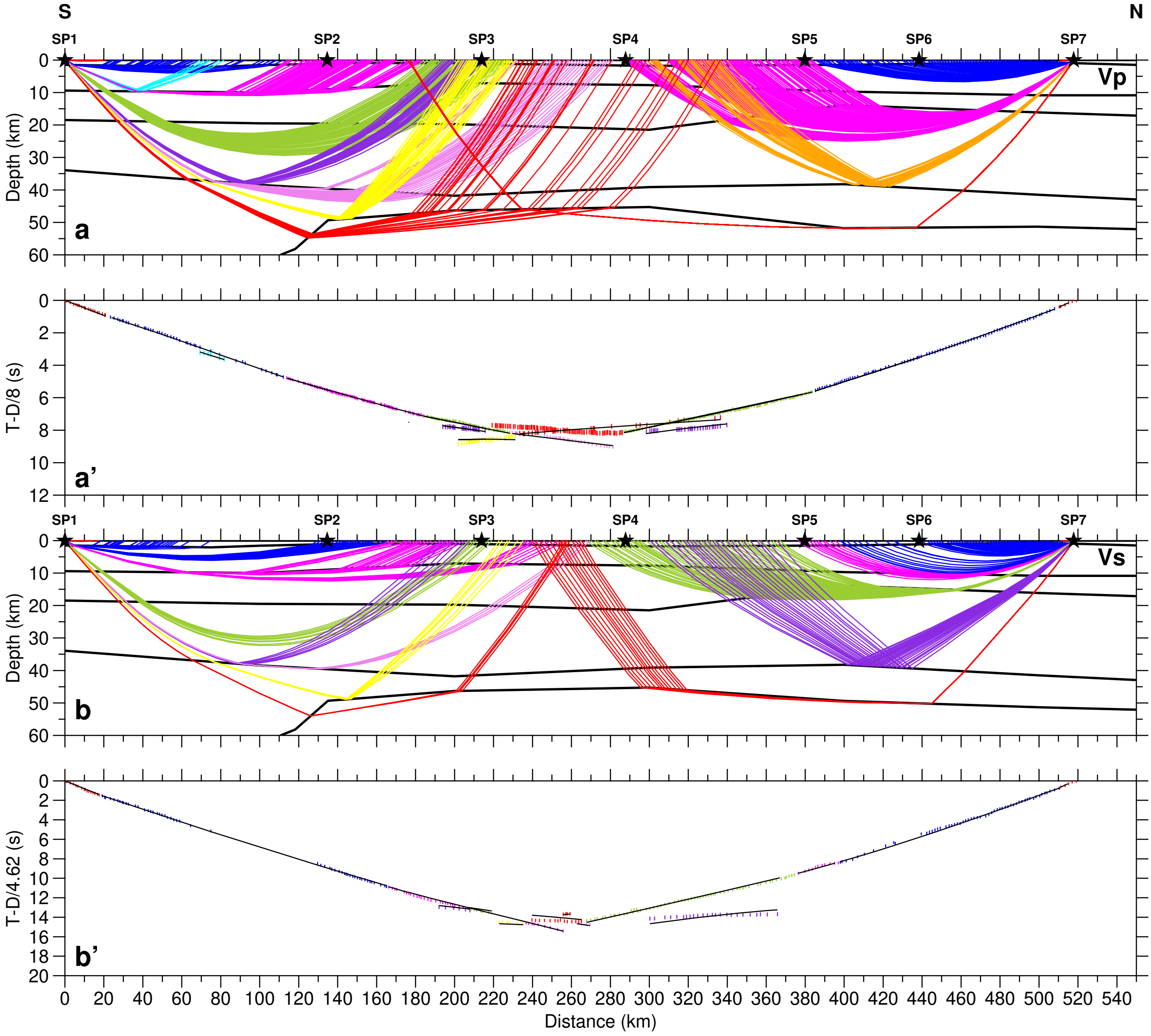

Supplementary Figure 1. Ray coverage and traveltime fit for SP1 and SP7. Ray tracing coverage of the seismic P- and S-wave model and P- and S-wave traveltime fit for the two end-shots SP1 and SP7, illustrating the high resolution of the lower crust and depth to Moho. Lines show calculated traveltimes and vertical bars show observed traveltimes with length of bar corresponding to uncertainty of pick

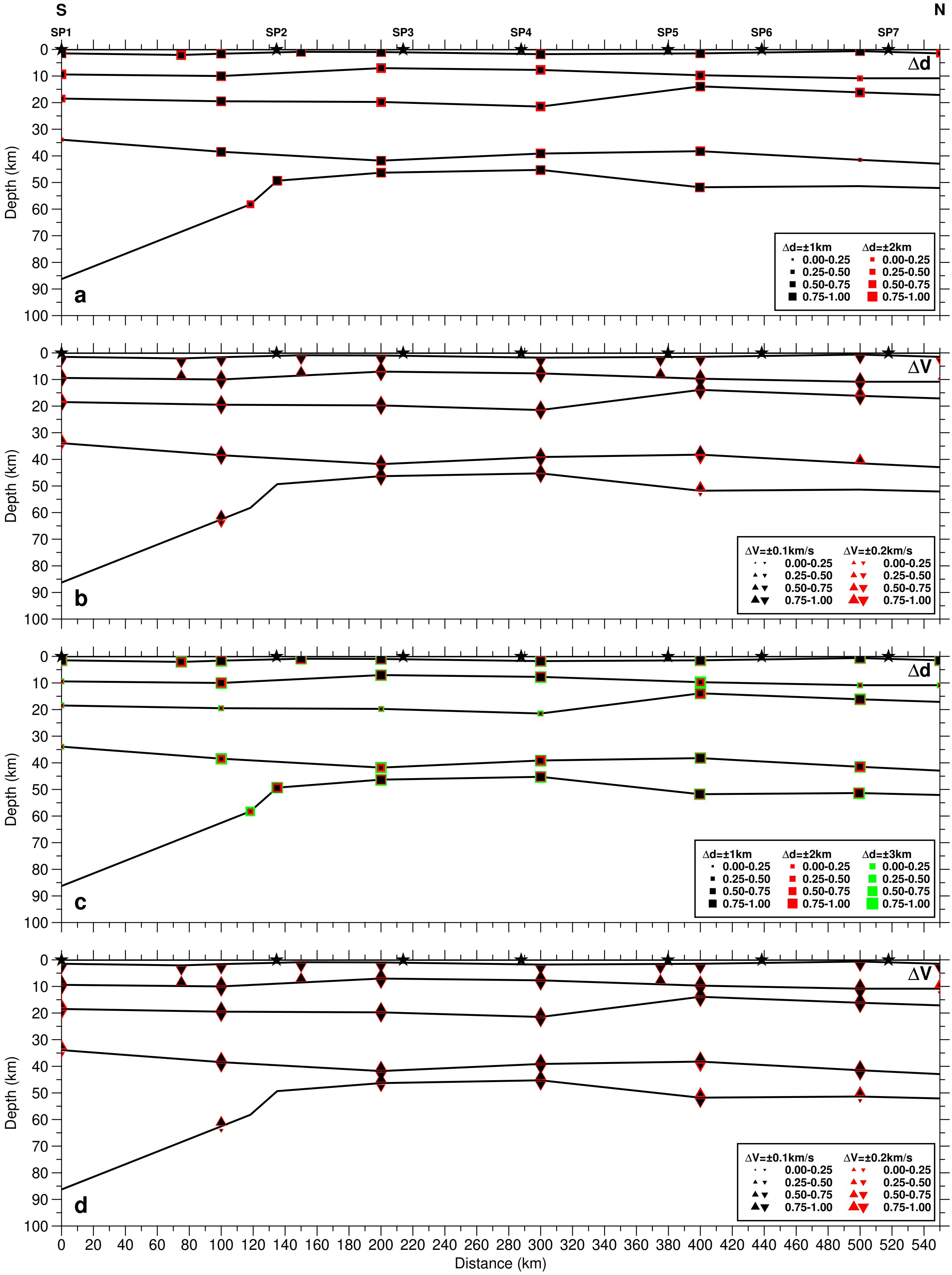

Supplementary Figure 2. Resolution test for the P- and S-wave velocity model. The diagonal values of the resolution matrix were calculated to test the model reliability for depths and velocities. (a) depth reliability (Δd=±1 and ±2 km) for the node depths of the P-wave model. (b) velocity reliability (ΔV=±0.1 and ±0.2 km/s) for the node velocities of the P-wave model. (c) depth reliability (Δd=±1, ±2 km and ±3 km) for the node depths of the S-wave model. (d) velocity reliability (ΔV=±0.1 and ±0.2 km/s) for the node velocities of the S-wave model.

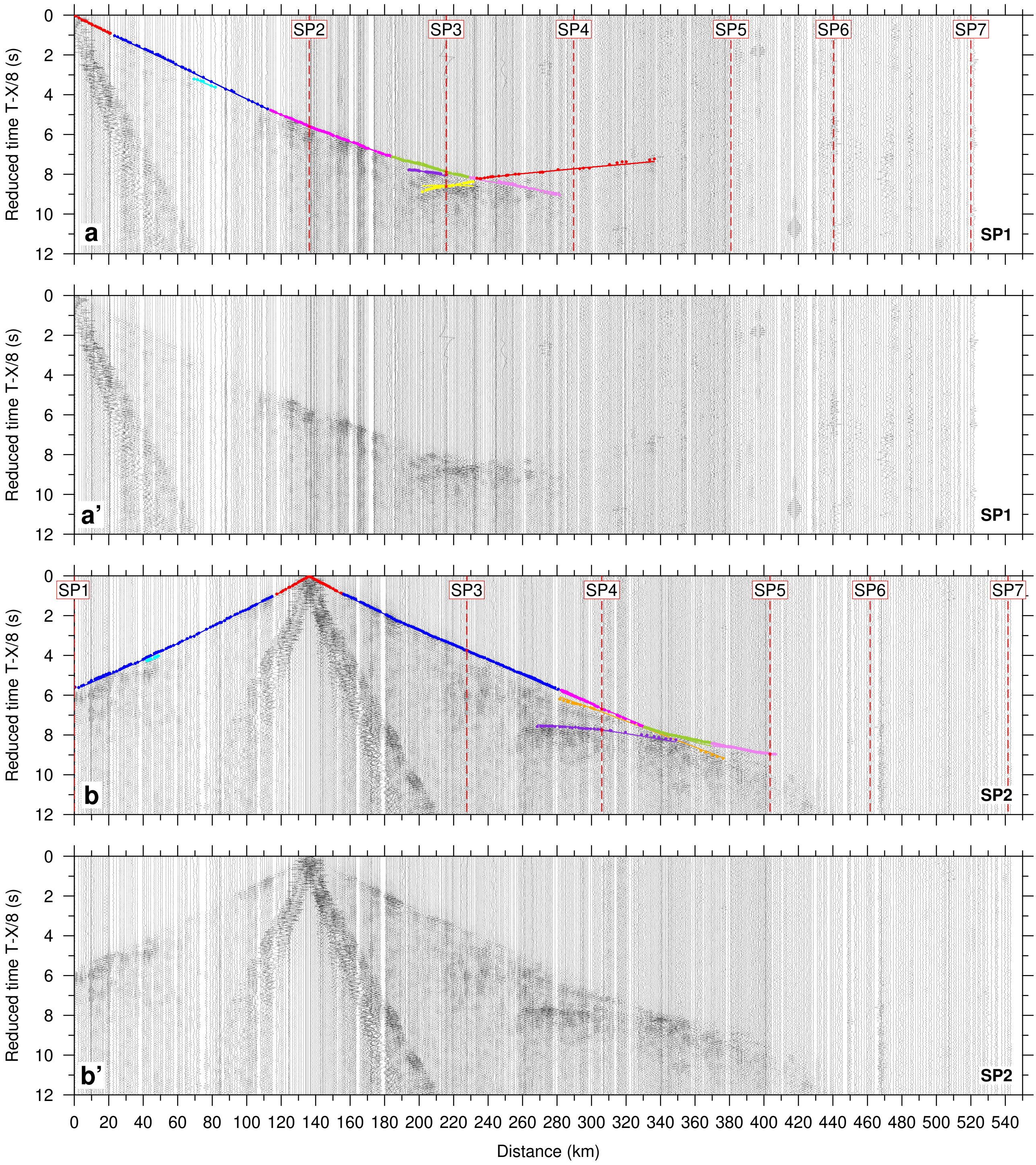

Supplementary Figure 3. Seismic sections for the seven shots (P-wave). The seismic sections include the picks (dots)and the traveltimes (solid lines) calculated for the final P-wave velocity model. The travel time is reduced by a velocity of 8.0 km/s. Locations of shot points along the profile are shown and reciprocal traveltimes are marked by red stars.

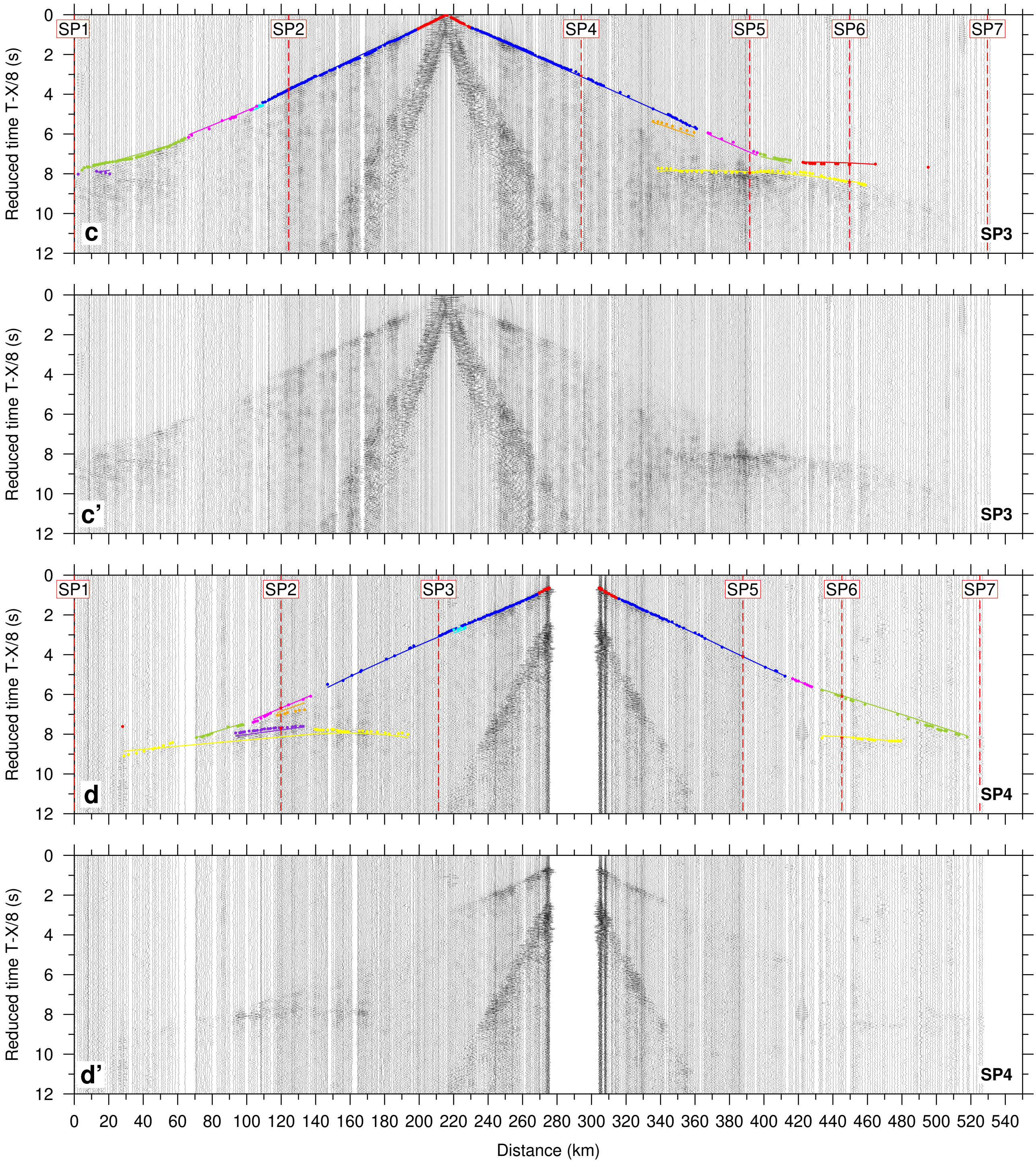

Supplementary Figure 3. Seismic sections for the seven shots (P-wave) (continued).

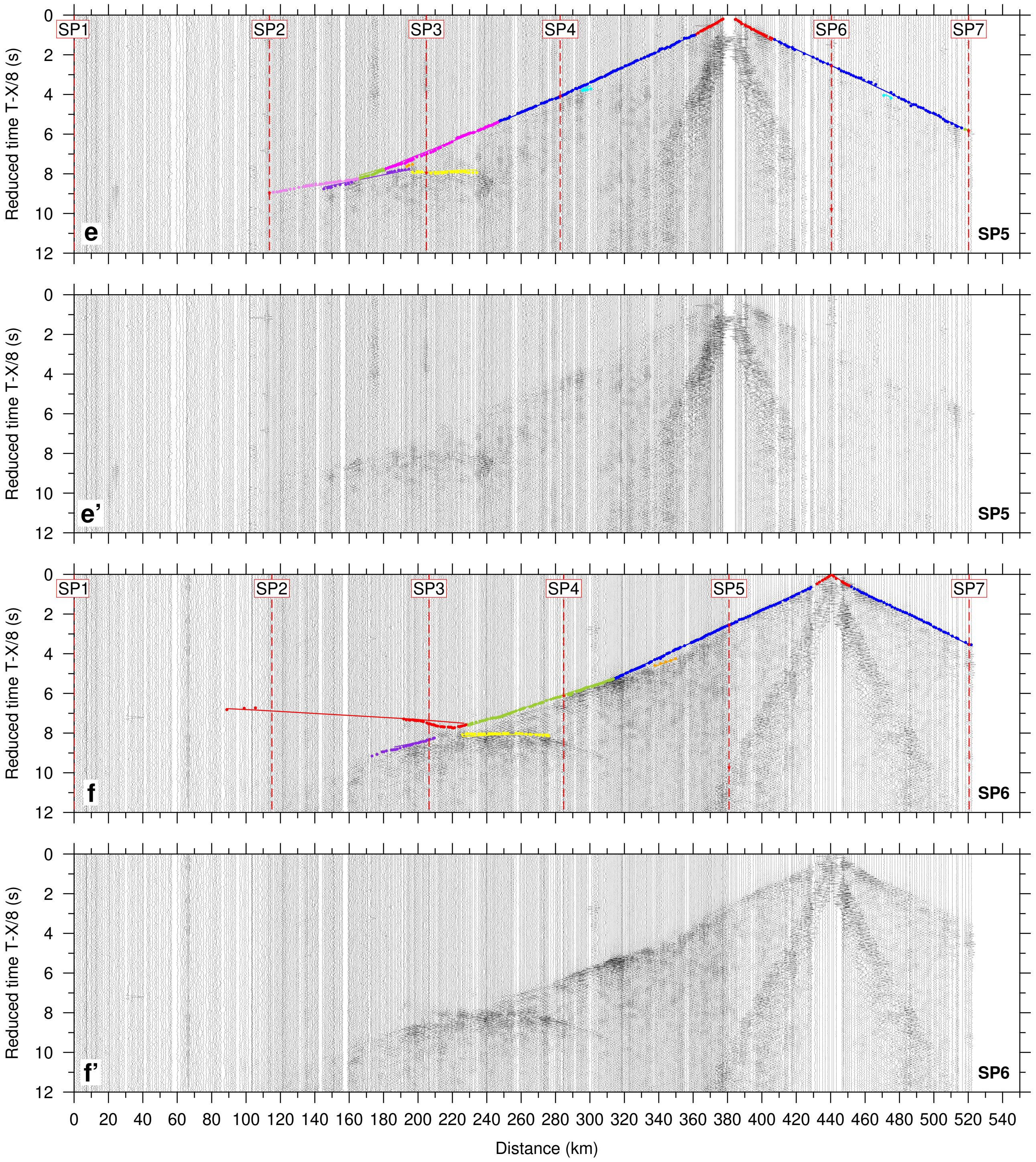

Supplementary Figure 3. Seismic sections for the seven shots (P-wave) (continued).

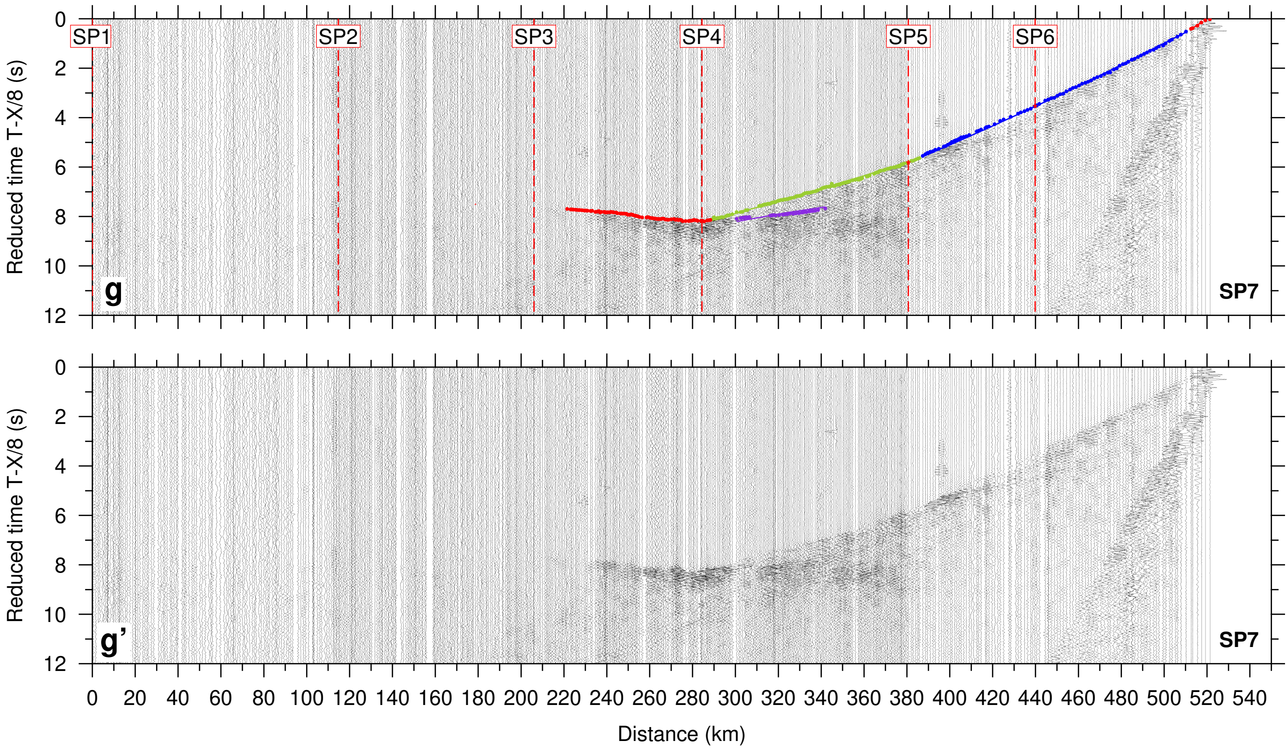

Supplementary Figure 3. Seismic sections for the seven shots (P-wave) (continued).

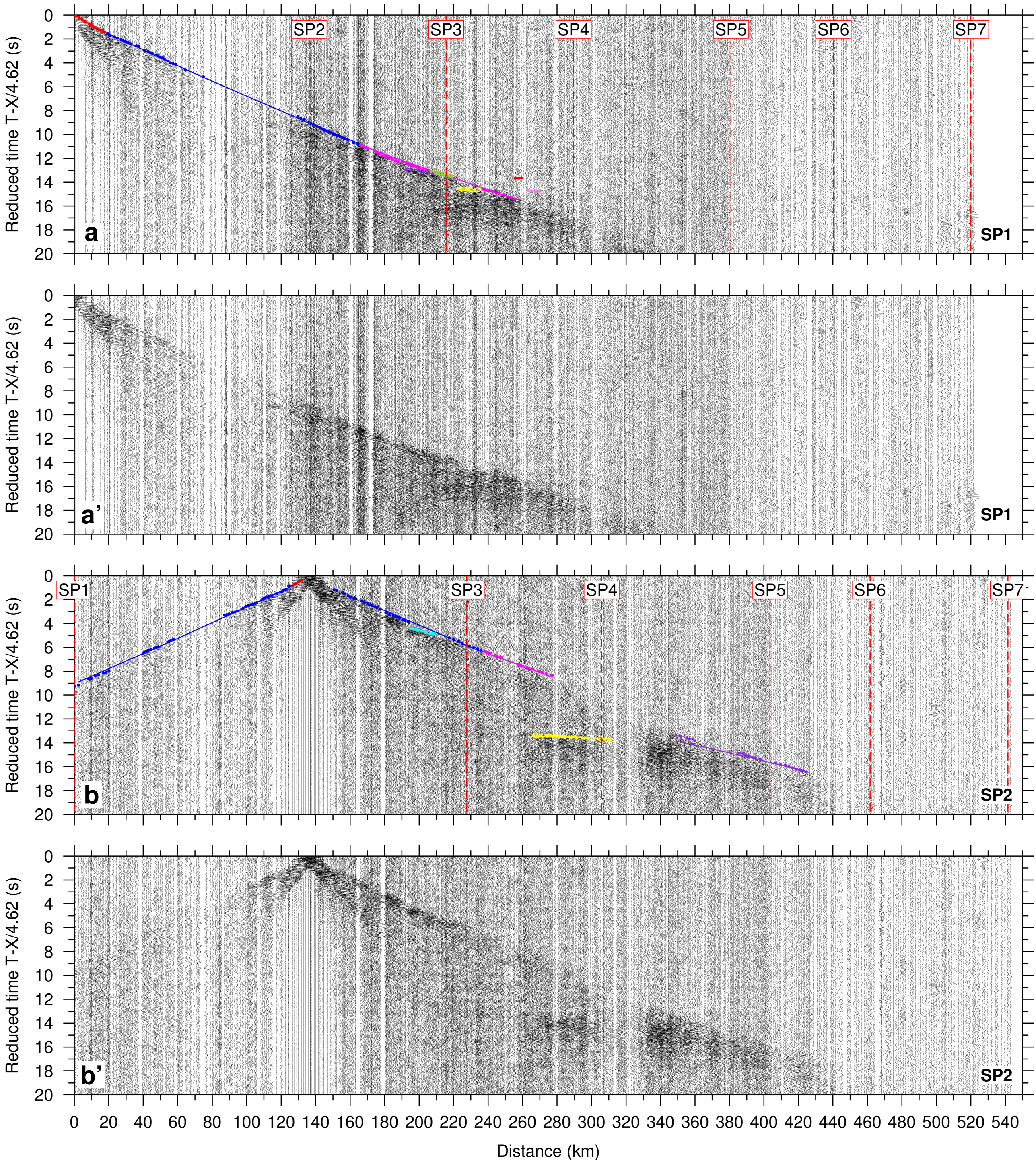

Supplementary Figure 4. Seismic sections for the seven shots (S-wave). The seismic sections include the picks (dots) and the traveltimes (solid lines) calculated for the final P-wave velocity model. The travel time is reduced by a velocity of 4.62 km/s. Locations of shot points along the profile are shown and reciprocal traveltimes are marked by red stars.

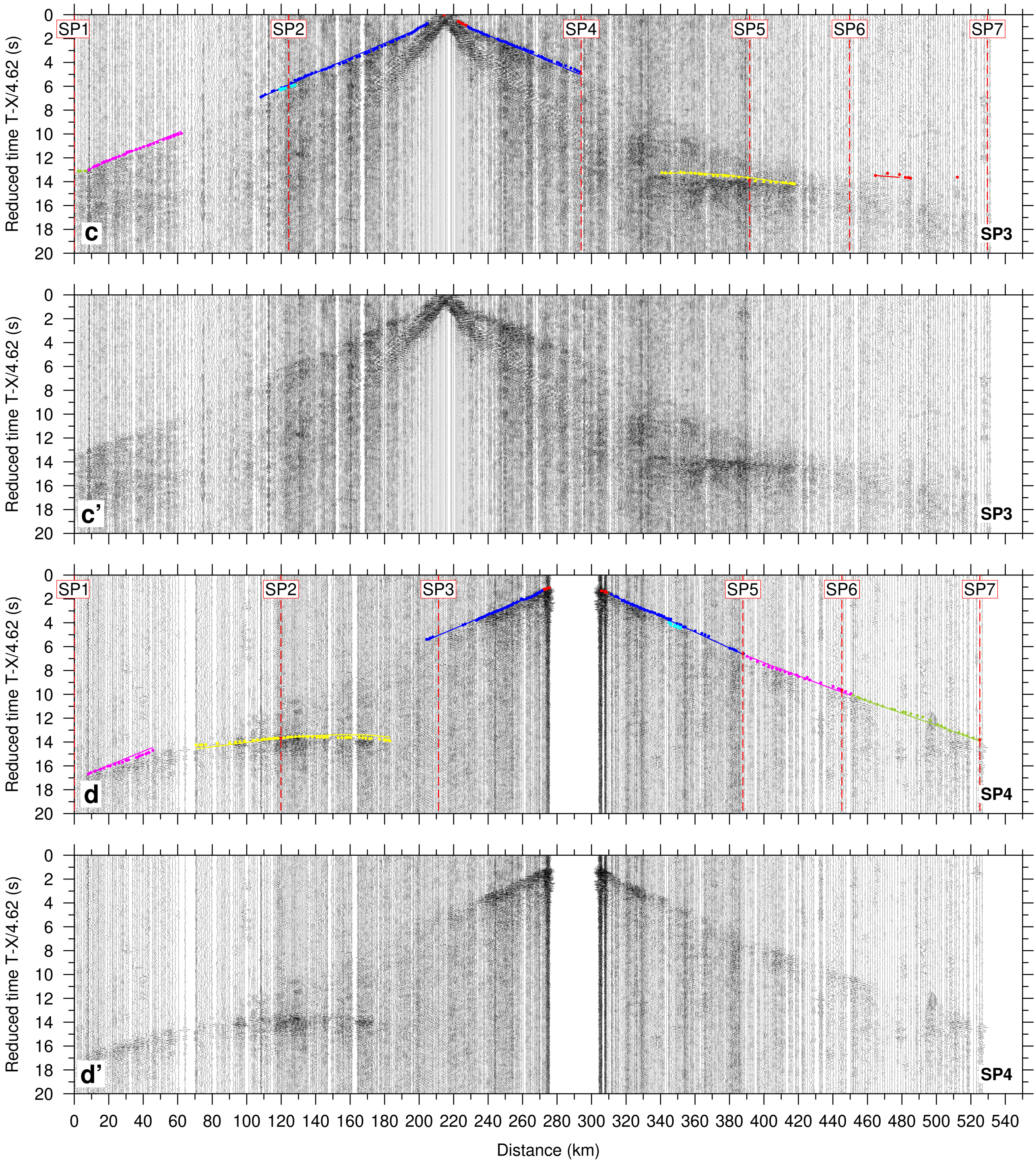

Supplementary Figure 4. Seismic sections for the seven shots (S-wave) (continued).

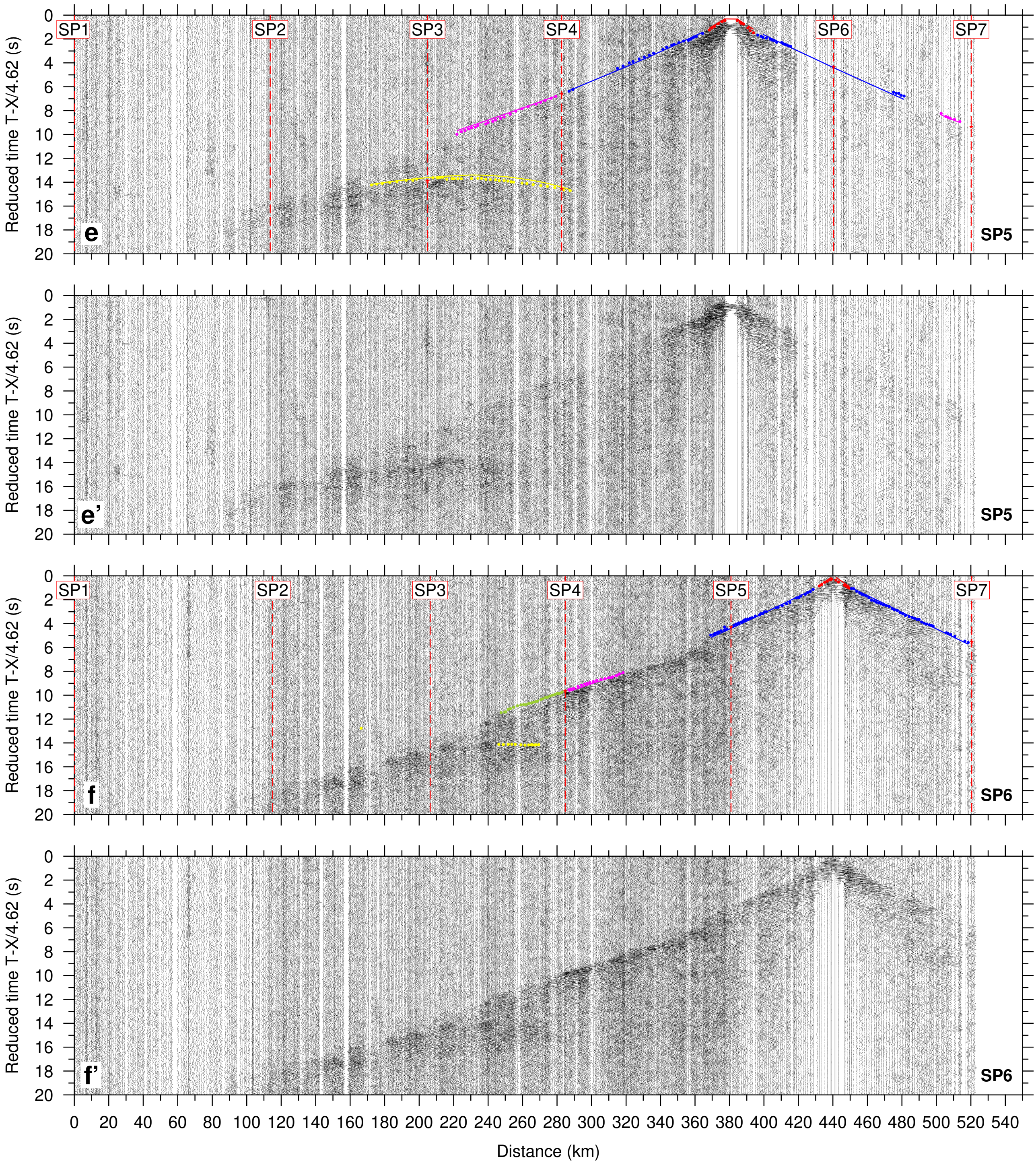

Supplementary Figure 4. Seismic sections for the seven shots (S-wave) (continued).

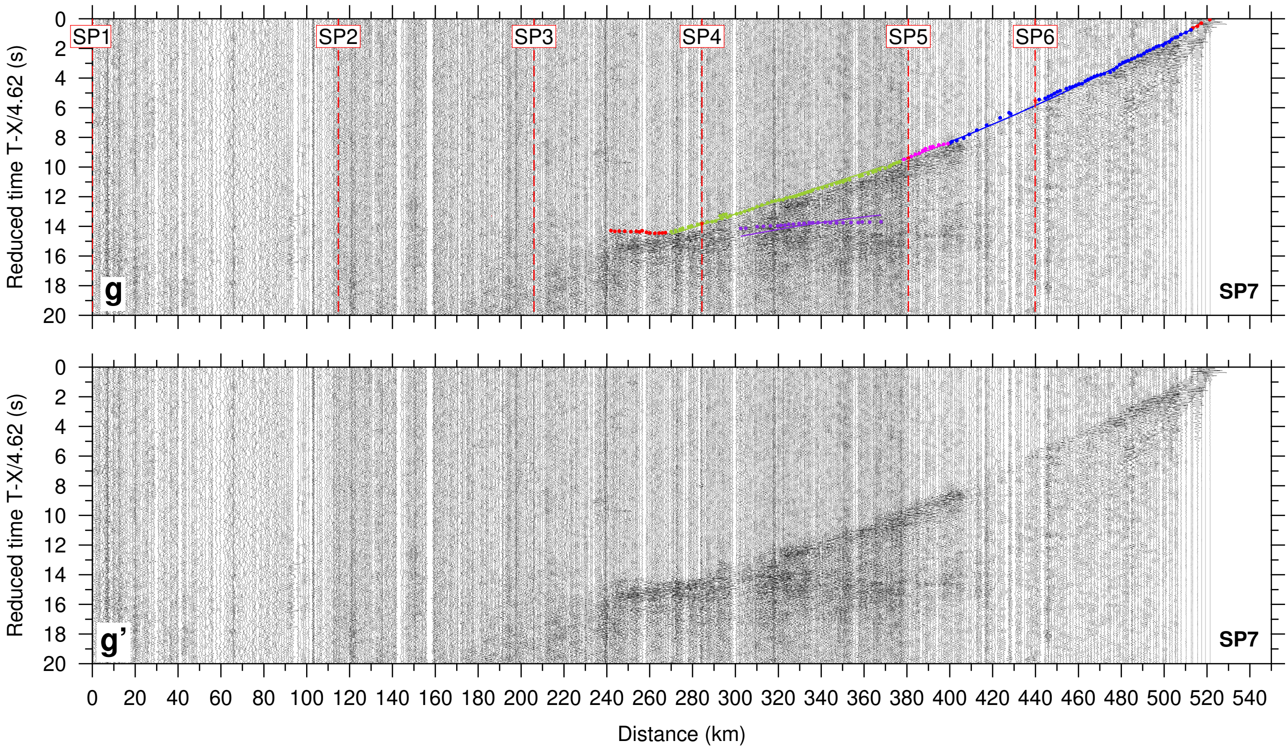

Supplementary Figure 4. Seismic sections for the seven shots (S-wave) (continued).

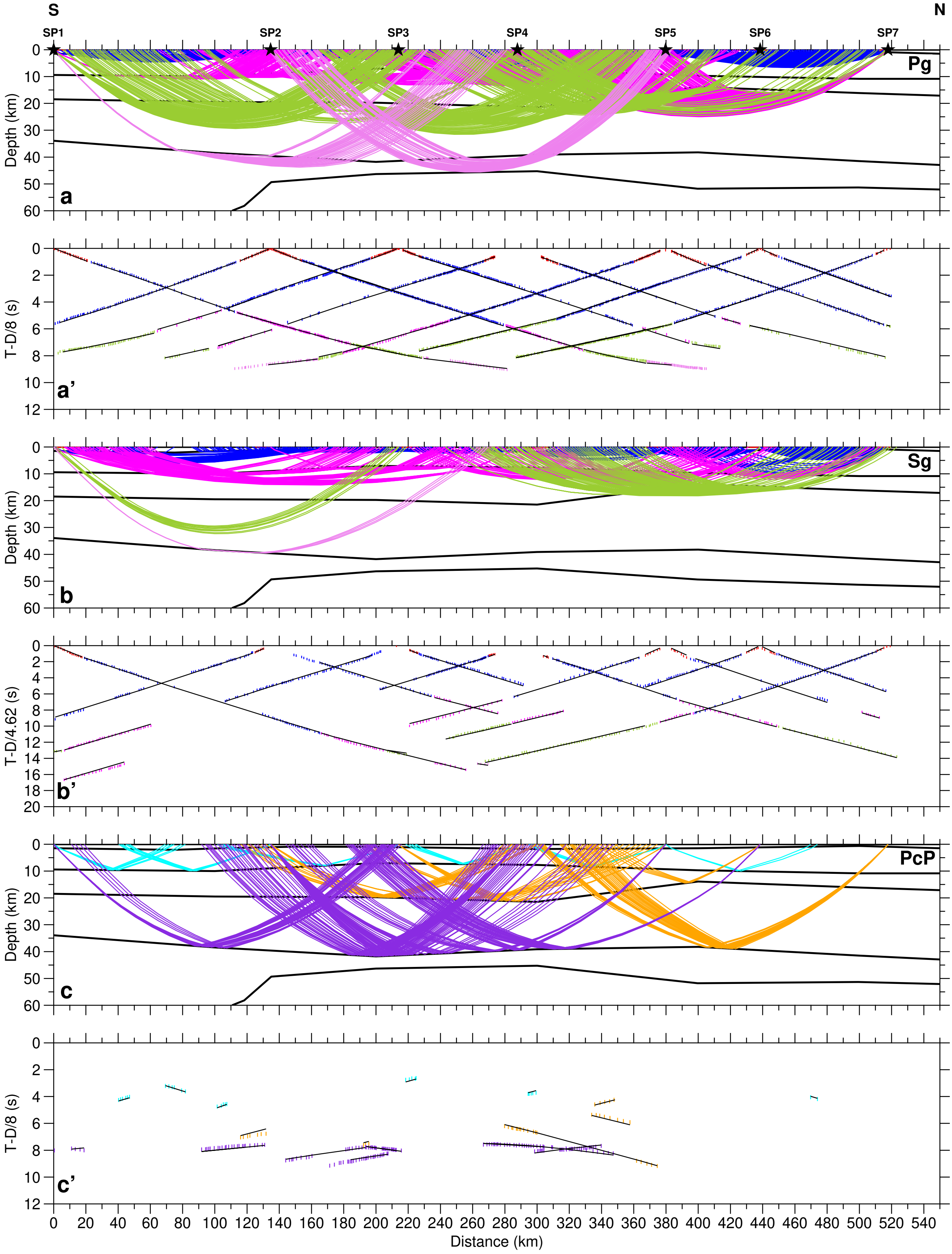

Supplementary Figure 5. Ray tracing results for different seismic phases. Ray tracing results calculated by the RAYINVR program. The upper panels show the rays, and the bottom panels show the picks (vertical bars of length corresponding to uncertainty) and the calculated times (solid lines) corresponding to the rays shown in the upper panels.

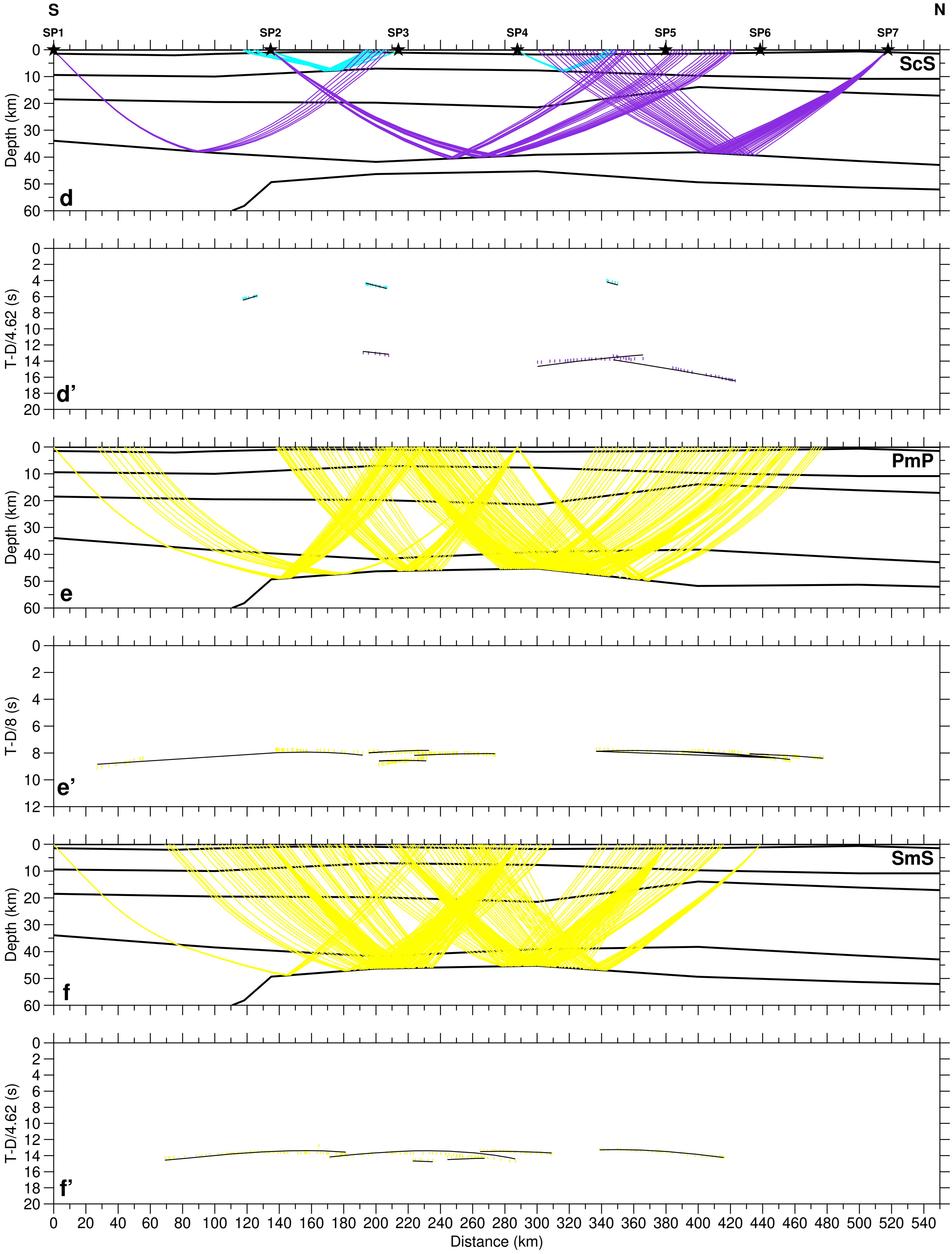

Supplementary Figure 5. Ray tracing results for different seismic phases (continued).

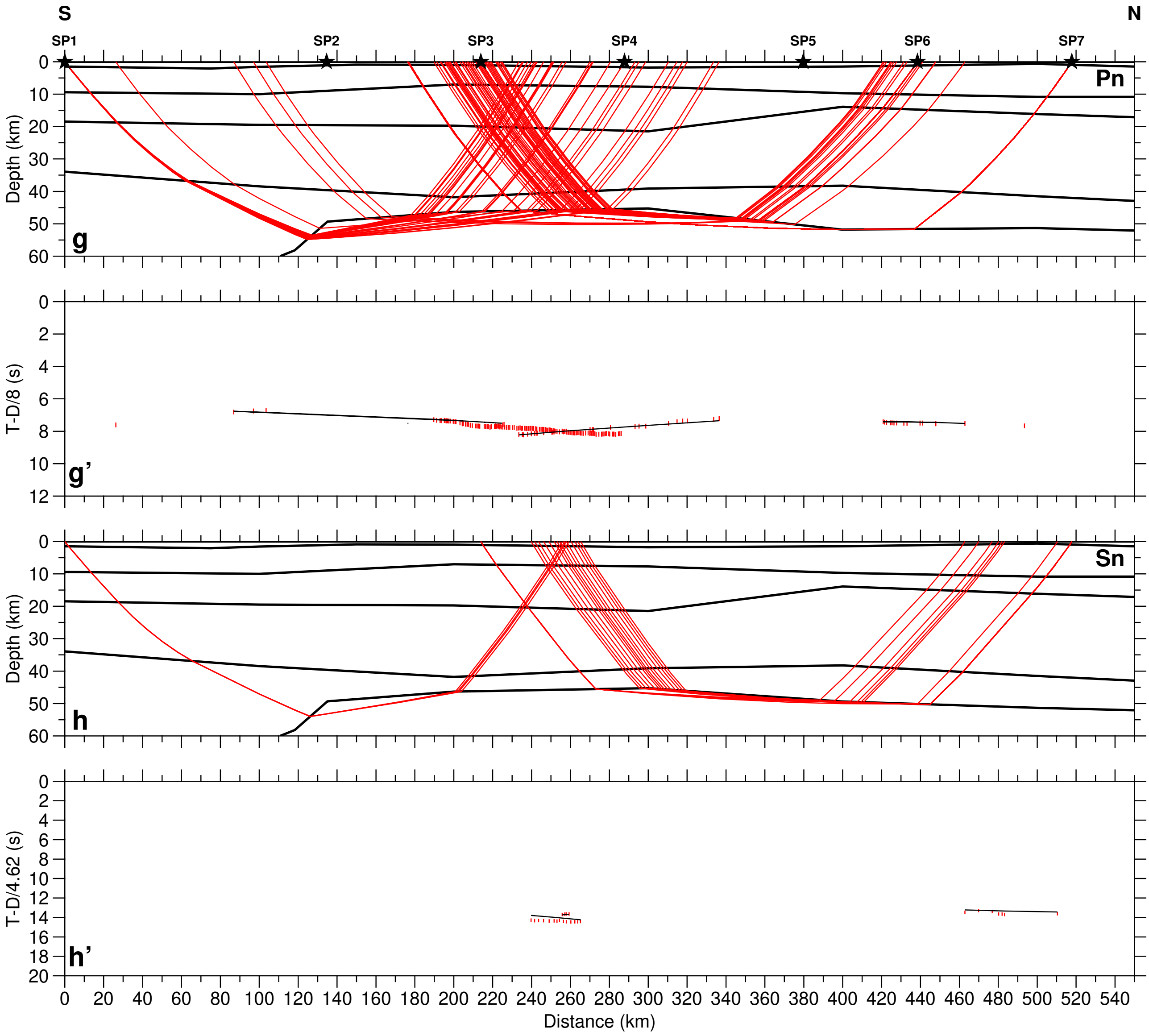

Supplementary Figure 5. Ray tracing results for different seismic phases (continued).

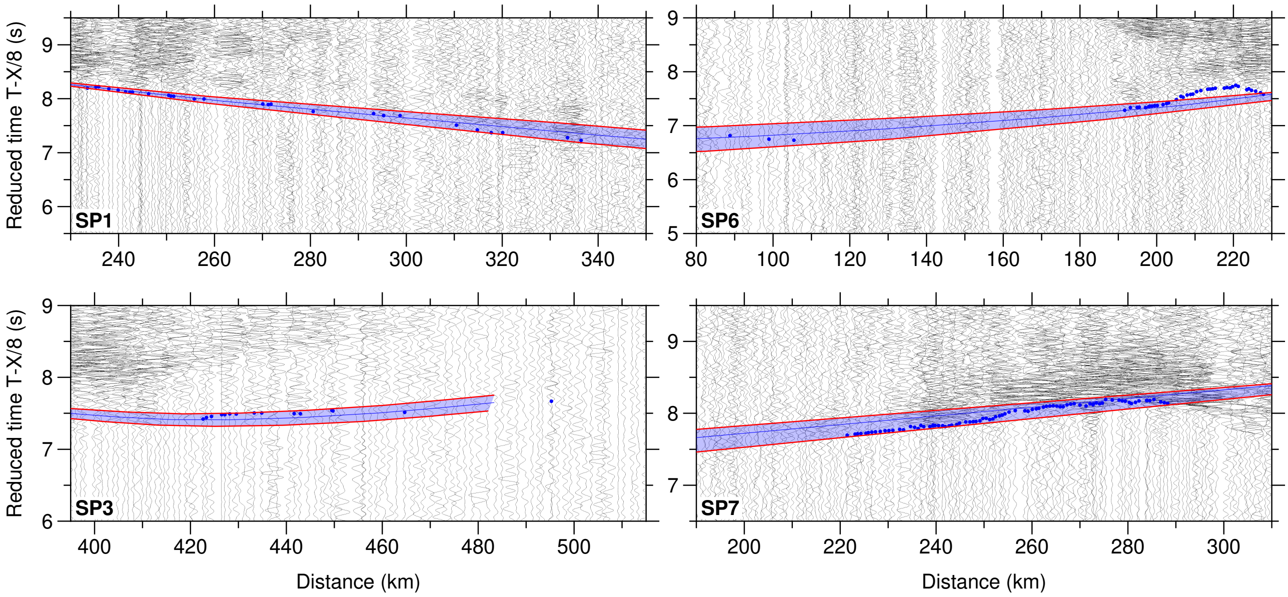

Supplementary Figure 6. Test of upper mantle velocity. The calculated arrival times of the Pn phases with different velocities in the upper mantle. The shaded zones between the top and bottom red lines show the arrival times between the velocity of 8.4 km/s (upper red lines) and 8.6 km/s (bottom red lines). The dots show the picked Pn arrival times.

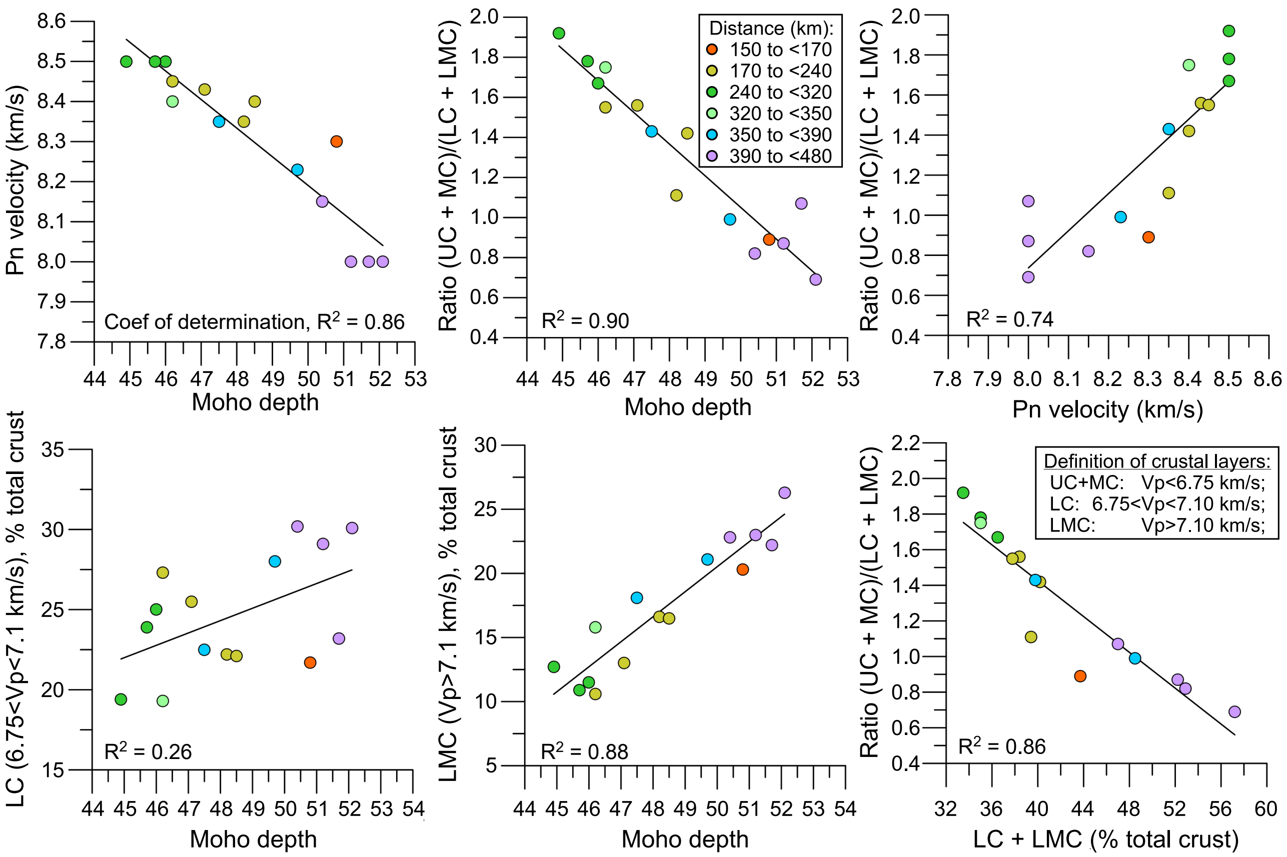

Supplementary Figure 7. X-Y plots of all parameters. Relations between moho depth, uppermost mantle Pn velocity, thickness of the lower crust, and ratio of the thicknesses of the upper-middle to mafic lower crust.

Supplementary Table 1. Seismic station parameters for data acquisition.

No. of stations	Recorder	Sampling rate [ms]	Geophone type
139	1C DATA-CUBE3	1.25	Spike 4.5 Hz
20	3C DATA-CUBE3	2.5	
263	Texan	4	
148	RAU 1C	1	
23	RAU 3C	1	DSU3 (MEMS)

Supplementary Table 2. Shot parameters for data acquisition.

Shot points Number	Latitude (°)	Longitude (°)	Altitude (m)	Time (UTC) y:d:h:m:s	No. of boreholes	TNT charge [kg]
SP1	58.0914	15.6291	165	2017:151:12:00:04.429	5	500
SP2	59.1123	16.9217	57	2017:151:03:05:12.976	3	360
SP3	59.9306	16.8074	91	2017:150:04:15:04.413	4	400
SP4	60.6320	16.7130	95	2017:152:12:07:04.662	11	475
SP5	61.4934	16.3262	253	2017:151:12:10:04.189	4	400
SP6	62.0237	16.4557	271	2017:150:21:10:04.710	4	400
SP7	62.7427	16.4366	322	2017:150:03:10:03.633	5	500

Supplementary Table 3. Statistical parameters of seismic P-wave modelling for different picks.

Phase number	Total number of picks	Number of picks for modelling	t_{RMS} (ms)	χ^2
Pg1	211	197	29	0.083
Pg2	1020	1011	42	0.181
Pc2P	29	29	84	0.323
Pg3	224	223	55	0.306
Pc3P	41	41	134	0.817
Pg4	420	418	68	0.459
Pc4P	160	152	80	0.285
Pg5	120	78	64	0.420
PmP	184	184	107	0.507
Pn	169	81	131	0.776
Total	2601	2414	65	0.303

Supplementary Table 4. Statistical parameters of seismic S-wave modelling for different picks.

Phase number	Total number of picks	Number of picks for modelling	t_{RMS} (ms)	χ^2
Sg1	70	67	107	0.517
Sg2	508	486	103	0.471
Sc2S	23	23	117	0.358
Sg3	184	183	128	0.735
Sg4	125	117	120	0.646
Sc4S	54	54	260	1.728
Sg5	4	4	74	0.324
SmS	157	156	208	1.421
Sn	25	7	143	0.779
Total	1150	1097	140	0.727

Supplementary References

1. Abalos, B., Fountain, D.M., Gil Ibarguchi, J.I. & Puelles, P. Eclogite as a seismic marker in subduction channels: seismic velocities, anisotropy, and petrofabric of Cabo Ortegal eclogite tectonies (Spain). *Geol. Soc. Am. Bull.* **123,** 439-456 (2011).

2. Austrheim, H. & Moerk, M.B.E. The lower continental crust of the Caledonian mountain chain: evidence from former deep crustal sections in western Norway. *Nor. Geol. Unders., Spec. Publ.* **3,** 102-113 (1988).

3. Austrheim, H. Eclogitization of lower crustal granulites by fluid migration through shear zones. *Earth and Planetary Science Letters* **81,** 221-232 (1987).

4. Austrheim, H. The granulite-eclogite facies transition: A comparison of experimental work and a natural occurrence in the Bergen Arcs, western Norway. *Lithosphere* **25,** 163-169 (1990).

5. Austrheim, H. Eclogite formation and dynamics of crustal roots under continental collision zones. *Terra Nova* **3,** 492-499 (1991).

6. Austrheim, H., Erambert, M., & Engvik, A. K. Processing of crust in the root of the Caledonian continental collision zone: The role of eclogitization. *Tectonophysics* **273,** 129-153 (1997).

7. Bascou, J., Barroul, G., Vauchez, A., Mainprice, D. & Egydio-Silva, M. EBSD-measured lattice-preferred orientations and seismic properties of eclogites. *Tectonophysics* **342,** 61-80 (2001).

8. Bascou, J. et al. Seismic velocities, anisotropy and deformation in Siberian cratonic mantle: EBSD data on xenoliths from the Udachnaya kimberlite. *Earth and Planetary Science Letters* **304,** 71-84 (2011).

9. Christensen, N. I. Poisson's ratio and crustal seismology. *J. Geophys. Res.* **101,** 3139-3156 (1996).

10. Fountain D. M., Austrheim H. & Boundy T. M. Compressional wave velocities across the garnet granulite- eclogite facies transition (Bergen Arcs, Norway). *Abstr. Am. Geophys. Un., Spring meeting* (1990).

11. Fountain, D. M., Boundy, T .M., Austrheim, H. & Rey, P. Eclogite-facies shear zones - deep crustal reflectors? *Tectonophysics* **232,** 411-424 (1994).

12. Green D. H. & Ringwood A. E. An experimental investigation of the gabbro to eclogite transformation and its petrological applications. *Geochimica et Cosmochimica Acta* **31,** 767-833 (1967).

13. Hacker B. R. Eclogite formation and the rheology, buoyancy, seismicity, and H_2O content of oceanic crust. in *Subduction: Top to Bottom* (eds. Bebout, G. E., Scholl, D. W., Kirby, S. H. & Platt, J. P.) 337-346 (AGU, Geophys. Monograph, 96, Washington, DC, 1996).

14. Hacker B. R. et al. Exhumation of ultrahigh-pressure continental crust in east central China: Late Triassic-Early Jurassic tectonic unroofing. *Journal of Geophysical Research* **105(B6),** 13339-13364 (2000).

15. Ji, S. et al. Microstructures, petrofabrics and seismic properties of ultra high-pressure eclogites from Sulu region, China: implications for rheology of subducted continental crust and origin of mantle reflections. *Tectonophysics* **370,** 49-76 (2003).

16. Ji, S., Wang, Q. & Xia, B. *Handbook of seismic properties of minerals, rocks and ores* (Polytechnique International Press, Montreal, 2002).

17. Kern, H., Gao, S., Jin, Z., Popp, T. & Jin, S. Petrophysical studies on rocks from the Dabie ultrahigh-pressure (UHP) metamorphic belt, Central China: implications for the composition and delamination of the lower crust. *Tectonophysics* **301,** 191-215 (1999).

18. Kobussen, A. F., Christensen, N. I. & Thybo, H. Constraints on seismic velocity anomalies beneath the Siberian craton from xenoliths and petrophysics. *Tectonophysics* **425,** 123-135 (2006).

19. Kopylova, M. G., Lo, J. & Christensen, N. I. Petrological constraints on seismic properties of the Slave upper mantle (Northern Canada). *Lithosphere* **77,** 493-510 (2004).

20. Manghnani, M. H., Ramananantandro, R. & Clark, S.P. Compressional and shear wave velocities in granulite facies rocks and eclogites to 10 kbar. *J. Geophys. Res.* **79,** 5427-5446 (1974).

21. Mauler, A., Burlini, L., Kunze, K., Philippot, P. & Burg, J. P. P-wave anisotropy in eclogites and relationship to the omphacite crystallographic fabric. *Phys. Chem. Earth* **25,** 119-126 (2000).

22. Moerk, M. B. E. A gabbro to eclogite transition on Flemsoy, Sunnmore, western Norway. *Chemical Geology* **50,** 283-310 (1985).

23. Richardson, S. W. & England, E. C. Metamorphic consequences of crustal eclogite production in overthrust orogenic zones. *Earth and Planetary Science Letters* **42,** 183-190 (1979).

24. Rudnick, R. L. & Fountain, D. M. Nature and composition of the continental crust: a lower crustal perspective. *Rev. Geophys.* **33,** 267-309 (1995).

25. Shulgin A., Faleide J. I., Mjelde R., Breivik A. & Huismans R. Crustal domains in the Western Barents Sea. *Geophys. J. Int.* **221,** 2155-2169 (2020).

26. Wang, Q., Burlini, L., Mainprice, D. & Xu, Z. Q. Geochemistry, petrofabrics and seismic properties of eclogites from the Chinese Continental Scientific Drilling boreholes in the Sulu UHP terrane, eastern China. *Tectonophysics* **475,** 251-266 (2009).

27. Worthington, J. R., Hacker B. R. & Zandt G. Distinguishing eclogite from peridotite: EBSD-based calculations of seismic velocities. *Geophys. J. Int.* **193,** 489-505 (2013).
